# Validating the Assumptions of Population Adjustment: Application of Multilevel Network Meta-regression to a Network of Treatments for Plaque Psoriasis

**DOI:** 10.1177/0272989X221117162

**Published:** 2022-08-23

**Authors:** David M. Phillippo, Sofia Dias, A. E. Ades, Mark Belger, Alan Brnabic, Daniel Saure, Yves Schymura, Nicky J. Welton

**Affiliations:** University of Bristol, Bristol, UK; University of Bristol, Bristol, UK; University of York, York, North Yorkshire, UK; University of Bristol, Bristol, UK; Lilly UK, Windlesham, Surrey, UK; Eli Lilly Australia Pty. Limited, Sydney, NSW, Australia; Lilly Deutschland GmbH, Bad Homburg, Hessen, Germany; Lilly Deutschland GmbH, Bad Homburg, Hessen, Germany; University of Bristol, Bristol, UK

**Keywords:** effect modification, indirect comparison, individual patient data, network meta-analysis, population adjustment

## Abstract

**Background:**

Network meta-analysis (NMA) and indirect comparisons combine aggregate data (AgD) from multiple studies on treatments of interest but may give biased estimates if study populations differ. Population adjustment methods such as multilevel network meta-regression (ML-NMR) aim to reduce bias by adjusting for differences in study populations using individual patient data (IPD) from 1 or more studies under the conditional constancy assumption. A shared effect modifier assumption may also be necessary for identifiability. This article aims to demonstrate how the assumptions made by ML-NMR can be assessed in practice to obtain reliable treatment effect estimates in a target population.

**Methods:**

We apply ML-NMR to a network of evidence on treatments for plaque psoriasis with a mix of IPD and AgD trials reporting ordered categorical outcomes. Relative treatment effects are estimated for each trial population and for 3 external target populations represented by a registry and 2 cohort studies. We examine residual heterogeneity and inconsistency and relax the shared effect modifier assumption for each covariate in turn.

**Results:**

Estimated population-average treatment effects were similar across study populations, as differences in the distributions of effect modifiers were small. Better fit was achieved with ML-NMR than with NMA, and uncertainty was reduced by explaining within- and between-study variation. We found little evidence that the conditional constancy or shared effect modifier assumptions were invalid.

**Conclusions:**

ML-NMR extends the NMA framework and addresses issues with previous population adjustment approaches. It coherently synthesizes evidence from IPD and AgD studies in networks of any size while avoiding aggregation bias and noncollapsibility bias, allows for key assumptions to be assessed or relaxed, and can produce estimates relevant to a target population for decision-making.

**Highlights:**

- Multilevel network meta-regression (ML-NMR) extends the network meta-analysis framework to synthesize evidence from networks of studies providing individual patient data or aggregate data while adjusting for differences in effect modifiers between studies (population adjustment). We apply ML-NMR to a network of treatments for plaque psoriasis with ordered categorical outcomes.
- We demonstrate for the first time how ML-NMR allows key assumptions to be assessed. We check for violations of conditional constancy of relative effects (such as unobserved effect modifiers) through residual heterogeneity and inconsistency and the shared effect modifier assumption by relaxing this for each covariate in turn.
- Crucially for decision making, population-adjusted treatment effects can be produced in any relevant target population. We produce population-average estimates for 3 external target populations, represented by the PsoBest registry and the PROSPECT and Chiricozzi 2019 cohort studies.

## Introduction

Health care decision making requires reliable estimates of relative treatment effects based, ideally, on high-quality randomized controlled trials (RCTs) comparing the treatments of interest, in a relevant target population. However, head-to-head RCTs between all relevant treatments are seldom available. Instead, comparisons are conducted using standard indirect comparison or network meta-analysis (NMA) methods using published aggregate data (AgD) from each study.^1–4^ These methods assume that any variables that interact with treatment (effect modifiers) are balanced between study populations and that the study populations are representative of the target population of interest, which may not always be the case. Recently, population adjustment methods have been proposed that allow this assumption to be relaxed by adjusting for differences in effect modifiers using available individual patient data (IPD) from 1 or more studies.^5–14^

**Figure 1:**
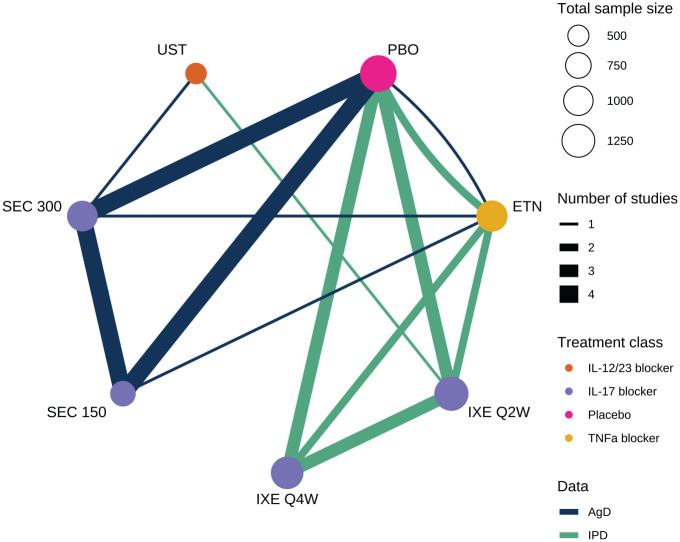
Network of studies comparing treatments for moderate-to-severe plaque psoriasis. PBO, placebo; IXE, ixekizumab; SEC, secukinumab; ETN, etanercept; UST, ustekinumab. IXE and SEC were each investigated with 2 different dosing regimens.

We use the motivating example of a network of 6 active treatments plus placebo for moderate-to-severe plaque psoriasis from a previous systematic review,^15^ shown in Figure 1. IPD on outcomes and baseline covariates are available from 4 studies, and AgD consisting of summary outcomes and baseline covariate summaries are available for the remaining 5 studies (Table A.1). Outcomes of interest include success/failure to achieve at least 75%, 90%, or 100% improvement on the Psoriasis Area and Severity Index (PASI) scale at 12 weeks compared to baseline, denoted PASI 75, PASI 90, and PASI 100, respectively.

Matching-adjusted indirect comparison (MAIC)^8,10^ and simulated treatment comparison (STC)^9,10^ use reweighting or regression adjustment, respectively, to adjust the results of an IPD study to the population of an AgD study and estimate a population-adjusted indirect comparison. However, these methods were designed for 2-study indirect comparisons (1 IPD and 1 AgD). Although extensions to indirect comparisons with more than 1 IPD or AgD study have been proposed,^16^ these methods do not generalize easily to larger networks of studies and treatments that are frequently encountered in practice.^17^ Warren et al.^15^ previously analyzed this network (Figure 1) using multiple MAIC analyses (following Belger et al.^16^), comparing ixekizumab every 2 weeks against secukinumab 300 mg via either etanercept or placebo and ustekinumab against secukinumab 300 mg via etanercept. However, these separate MAIC analyses are not coherent, in the same way that performing multiple pairwise meta-analysis does not ensure coherent estimates unlike those produced from a NMA.^18^ Furthermore, these multiple analyses are not independent, as they reuse the same data, and none of them use all available data (Table A.1). Crucially, unless further assumptions are made, MAIC and STC are limited to producing estimates for the population of the AgD study, which may not match the target population for a decision.^11,12^ The AgD studies included in each of these analyses also differ, meaning that each analysis produces results for different target populations and thus are not directly compatible.

In the wider meta-analysis literature, several authors have considered combining both the IPD and AgD in network meta-regression models to support the estimation of effect modifier interactions and increase statistical power and precision.^5–7^ However, these methods simply “plug in” mean covariate values for the aggregate studies, which results in aggregation bias in nonlinear models.^19^ Approaches that avoid this bias have been developed^20^ but are applicable only when all covariates are binary.

Multilevel network meta-regression (ML-NMR) has recently been proposed to address many of the limitations of previous approaches.^13,14^ ML-NMR works by defining an individual-level regression model, which is fitted directly to the individuals in the IPD studies, and incorporates summary outcomes from the AgD studies by integrating this individual-level model over the covariate distribution in each AgD study. As a result, ML-NMR appropriately synthesizes networks of IPD and AgD studies of any size, adjusting for differences in effect modifiers while avoiding aggregation bias and noncollapsibility bias. Crucially for decision making, ML-NMR can produce estimates of quantities of interest in any chosen target population, such as population-average treatment effects or absolute event probabilities in the decision target population. Furthermore, given sufficient data, ML-NMR may allow key assumptions to be assessed or relaxed.^13^ ML-NMR is an extension of the standard NMA framework, reducing to standard AgD NMA when no covariates are adjusted for and to full-IPD network meta-regression (the “gold standard” approach) when IPD are available from every study.^13^

The key assumption made by all population adjustment methods in connected networks is that there are no unobserved effect modifiers, so that the relative effects are constant given the effect modifiers adjusted for—the conditional constancy of relative effects assumption.^11,12^ In sufficiently large networks, this assumption may be assessed by checking for residual heterogeneity and inconsistency.^4,13,21^ ML-NMR has previously been applied to a subset of the plaque psoriasis network consisting of only 4 studies, considering only the PASI 75 outcome.^13^ However, no tests for heterogeneity or inconsistency were conducted. Furthermore, a shared effect modifier assumption^11^ between ixekizumab and secukinumab (meaning that the effect modifier interaction parameters were assumed common for these treatments) was required to identify the model. Lastly, while higher PASI outcomes are more clinically meaningful, low numbers of observed events for PASI 90 and PASI 100 pose difficulties for estimation in stand-alone analyses of each PASI outcome.

In this article, we demonstrate how the assumptions made by ML-NMR can be assessed in practice to obtain reliable treatment effect estimates in a target population. We jointly analyze the 3 PASI outcomes in an ordered categorical model, allowing information to be shared between outcomes and aiding estimation for the higher PASI outcomes. We show how to assess key assumptions using ML-NMR, including the conditional constancy of relative effects and shared effect modifier assumptions, which are untestable assumptions when using methods such as MAIC and STC. We produce estimates of population-average treatment effects and response probabilities for target populations represented by each of the studies in the network and for 3 external target populations.

## Example: Plaque Psoriasis

A previous systematic review found 9 studies comparing ixekizumab every 2 weeks or every 4 weeks, secukinumab at 150 mg dose or 300 mg dose, ustekinumab at a weight-based dose, and etanercept, along with placebo.^15^ These studies form the network in Figure 1 (summarized in Table A.1). IPD consisting of individual outcomes and baseline covariates were available from 4 studies, and AgD consisting of summary outcomes and baseline covariate summaries were extracted from published study reports on the remaining 5 studies (Table A.1). Table A2 summarizes the baseline covariate distributions in each of the 9 studies. We jointly analyze the PASI 75, PASI 90, and PASI 100 outcomes at 12 weeks. We consider adjustments for duration of psoriasis, previous systemic treatment, body surface area covered, weight, and psoriatic arthritis, which were considered potential effect modifiers based on expert clinical opinion in previous analyses.^13,22^ In a decision-making context such as health technology assessment, we typically require principled selection of effect modifiers prior to analysis, either through expert opinion, systematic review, or quantitative analyses of external evidence.^11^ Trials are typically underpowered to detect treatment-covariate interactions, and so selection based on post hoc criteria such as model fit, estimated effect size, or uncertainty may result in bias due to overinterpretation of chance findings or omission of true effect modifiers. Sensitivity analyses may be performed, for example, to compare the best-fitting model to the prespecified analysis model.

To be relevant for decision making, estimates must be produced for a decision target population. Typically, the decision target population is not best represented by any of the RCTs in the network; instead, such a target population may be chosen based on expert clinical knowledge or may be represented by a suitable registry or cohort study.^11^ To illustrate, we produce population-adjusted estimates for 3 external target populations (Table A.3).^23–26^

## Methods

Phillippo et al.^13^ introduced the general ML-NMR model, which we extend here for ordered categorical outcomes following the approach taken for AgD NMA.^4,27^

### ML-NMR for Ordered Categorical Outcomes

At the individual level, we have ordered categorical outcomes: <75% reduction in PASI score, ≥75% and <90% reduction, ≥90% and <100% reduction, and 100% reduction. These data are modeled using an ordered categorical likelihood, with the linear predictor for individual i in study j receiving treatment k given by

(1) $$\eta_{jk}(x_{ijk})=\mu_{j}+x_{ijk}^{T}(\beta_{1}+\beta_{2,k})+\gamma_{k},$$

which includes study-specific intercepts $\mu_{j}$ , individual-level covariates $x_{ijk}$ with main effects $\beta_{1}$ and treatment-covariate interactions $\beta_{2,k}$ (corresponding to prognostic and effect modifying terms, respectively), and individual-level treatment effects $\gamma_{k}$ (full details are given in Appendix A.1.1). Consistency is assumed for both the individual-level treatment effects and the interactions.^13^ We use the probit link function here for comparability with previous analyses, but the logit would be another suitable choice.

At the aggregate level, outcomes are vectors of summary outcome counts in each category. These summary data are given an ordered multinomial likelihood, with average event probabilities in each category obtained by integrating the individual-level model over the covariate joint distribution in each arm of each AgD study. We calculate these integrals numerically using quasi–Monte Carlo integration^13^ with 1000 integration points. To perform this integration, we require the covariate joint distributions in each of the AgD studies; however, these are often not directly available: typically, only marginal covariate information (e.g., means and standard deviations for continuous covariates, proportions for discrete covariates) is available from the AgD studies. We discuss how the covariate joint distributions can be reconstructed under some additional assumptions in the following section.

ML-NMR is typically implemented within a Bayesian framework. In this analysis, we place vague $N(0,10^{2})$ prior distributions on each of the model parameters, except for the latent ordered cutpoints, which are given improper uniform prior distributions U(−∞,+∞) .

### Using Published Marginal Covariate Information

To derive the aggregate-level model for ML-NMR through integration, the covariate joint distribution in each study is required. However, this is typically not available; instead, we have published marginal summary statistics for each covariate. We therefore reconstruct the full joint distribution in each AgD study by making assumptions regarding the forms of the marginal distributions and the correlation structure.^13^ Simulation studies have shown that these assumptions, even when incorrect, may have very little impact on the results in practice.^28^

In the plaque psoriasis analysis, marginal distributions for each covariate in the AgD studies are chosen to match the reported summary statistics, based on theoretical properties and the observed distributions in the IPD studies: weight and duration are given a Gamma distribution to account for skewness, and body surface area as a percentage is given a scaled logit-Normal distribution. Previous systemic treatment and psoriatic arthritis are binary covariates. The correlation matrix for the covariates in the AgD studies is set to equal the weighted average of the correlation matrices in the IPD studies.

### Checking Model Assumptions

The key assumption made by all population adjustment methods in connected networks is that there are no unobserved effect modifiers, so that the relative effects are constant given the effect modifiers adjusted for—the conditional constancy of relative effects assumption.^11,12^ This assumption implies that there is no remaining heterogeneity or inconsistency after adjusting for the given effect modifiers. In pairwise indirect comparisons, this is an untestable assumption; however, in larger networks, it is possible to assess this assumption by checking for residual heterogeneity and inconsistency,^13^ with techniques from standard AgD NMA.^4,21^

#### Assessing residual heterogeneity

We assess residual heterogeneity using a random effects (RE) ML-NMR model, in which the linear predictor $\eta_{jk}(x)$ in equation (1) is replaced by

(2a) $$\eta_{jk}(x)=\mu_{j}+x^{T}(\beta_{1}+\beta_{2,k})+\delta_{jk}$$

(2b) $$\delta_{jk}\sim N(\gamma_{k},\tau^{2})$$

(2c) $$\mathrm{cor}(\delta_{\mathrm{ja}},\delta_{\mathrm{jb}})=0.5$$

where $\delta_{j1}=0$ . The study-specific relative effects $\delta_{jk}$ within each study are multivariate Normal, with 0.5 correlations between non-treatment 1 arms under the assumption of common heterogeneity variance $\tau^{2}$ .^2^ Two-arm studies against treatment 1 have a single univariate Normal RE on the non-treatment 1 arm. We place a weakly informative half-Normal prior distribution $\mathrm{half}-N(0,2.5^{2})$ on the heterogeneity standard deviation τ , which means that there is 95% prior probability that τ lies between 0 and 5; τ=5 corresponds to 95% of true probit differences varying by ±10 between each study. Evidence of residual heterogeneity is assessed by comparing model fit under the fixed (FE) and RE models using the deviance information criterion (DIC)^29^ and examining the posterior distribution of τ .

#### Assessing residual inconsistency

We assess residual inconsistency using an unrelated mean effects (UME) ML-NMR model,^13,21^ where the linear predictor $\eta_{jk}(x)$ in equation (1) is replaced by

(3) $$\eta_{jk}(x)=\mu_{j}^{\left(t_{j1}\right)}+x^{T}(\beta_{1}+\beta_{2,k})+\gamma_{t_{j1}k},$$

where $t_{j1}$ is the treatment in arm 1 of study j and we set $\gamma_{\mathrm{kk}}=0$ for all k . We place independent vague $N(0,10^{2})$ prior distributions on each of the $\gamma_{\mathrm{ab}}$ parameters, which now represent independent and unrelated treatment contrasts (instead of imposing the consistency equations). Under the UME model, the study baselines $\mu_{j}^{\left(t_{j1}\right)}$ are now with respect to the treatments $t_{j1}$ in arm 1 of each study, rather than the network reference treatment 1. An RE UME model can also be fitted, replacing $\gamma_{t_{j1}k}$ with $\delta_{jt_{j1}k}$ in equation (3), which has a multivariate Normal structure analogous to that in equation (2).^13^ Evidence of residual inconsistency is assessed by comparing the overall model fit under the consistency model and the UME inconsistency model using the DIC and by comparing the residual deviance contributions from each data point under either model. If RE models are fitted, the heterogeneity standard deviation τ should also be compared, because a reduction in estimated heterogeneity under the inconsistency model can indicate the presence of inconsistency.^21^ As Donegan et al.^30^ described, inconsistency can also be present in the effect modifier interactions $\beta_{2,k}$ , which may be assessed through a similar approach to the UME model by placing independent prior distributions on the interactions on each contrast $\beta_{2,\mathrm{ab}}$ . However, there are insufficient data to assess inconsistency in the interaction terms in this network.

#### Assessing the shared effect modifier assumption

Estimation of the effect modifier interaction terms is data intensive, requiring IPD or sufficiently many AgD studies with different covariate distributions on each treatment.^13^ In the plaque psoriasis network, we have only 5 AgD studies that include secukinumab arms, which is not sufficient to simultaneously estimate independent interactions for every effect-modifying covariate. Instead, we make the shared effect modifier assumption^11,12^ for ixekizumab and secukinumab, both of which act as interleukin-17 blockers, meaning that these treatments share interaction terms and data requirements are shared across the class of treatments. That is, we set $\beta_{2,k}=\beta_{2,\mathrm{IL}-17}$ for each treatment k in this class. We assess this assumption directly, one covariate at a time, by splitting the common class interaction parameter for the covariate in question into independent treatment-specific interactions, while maintaining the common class interaction for the other covariates. Mathematically, for one covariate $l^{*}$ , in turn we let $\beta_{2,k;l^{*}}$ be independent for each treatment k , whereas the remaining covariates l retain the common class coefficients $\beta_{2,k;l}=\beta_{2,\mathrm{IL}-17;l}$ for each treatment k . We then compare the posterior distributions of the interaction estimates from the independent interaction model to the corresponding common interaction estimate from the model making the shared effect modifier assumption and compare the overall model fit using the DIC. If residual heterogeneity or inconsistency are detected, then these may be assessed again for the independent interaction models to determine whether an invalid shared effect modifier assumption was contributing to heterogeneity or inconsistency.

### Producing Population-Average Estimates for Populations of Interest

Population-average estimates of quantities of interest to decision making, such as average treatment effects and average event probabilities, can be produced by averaging estimates of individual-level quantities over the covariate joint distribution in the target population (see details in Appendix A.1.2).^13^ For decision making based on cost-effectiveness models, the typical inputs are the population-average event probabilities for a cohort-based model (e.g., a decision tree or Markov model) or individual event probabilities in the population for an individual-based model (e.g., a discrete event simulation). The target population need not be one of the studies in the network; indeed, it is more likely that it is best represented by a registry or cohort study.^11^

We produce estimates of population-adjusted average treatment effects in the 3 external target populations represented by the PsoBest registry^23,24^ and the PROSPECT^25^ and Chiricozzi 2019^26^ cohort studies. Following the process described in Appendix A.1.2, we require only the covariate summaries reported in Table A.3. Chiricozzi 2019 is the most different to the RCT populations, in patient age, body weight, disease severity, duration, and body surface area involvement, but there is still substantial overlap with the RCTs in the network, so extrapolation is limited. In the absence of available data on covariate correlations in the external target populations, we use the weighted average correlation matrix computed from the IPD studies.

We also produce population-adjusted average event probabilities in the external target populations. In addition to the covariate summaries, this requires information on the response rates on one treatment (active or placebo) in the target population, to inform the baseline risk. PASI 75 response counts on secukinumab 300 mg at 12 weeks are available from PROSPECT and Chiricozzi 2019, from which we construct Beta distributions for the average PASI 75 event probability on secukinumab 300 mg in each target population, Beta(1156, 1509–1165) and Beta(243, 330–243) respectively, that serve as a reference against which the remaining population-average event probabilities on each treatment in each of these external target populations are produced (following Appendices A.1.2 and A.1.3). No information on event rates was available from PsoBest.

### Statistical Software

All analyses were carried out in R version 4.0.2,^31^ using the package multinma.^32^ Models are estimated in a Bayesian framework using Stan.^33^

We fit the ML-NMR models described above, and for comparison, we also fit standard AgD NMA models with no covariate adjustment. For all models, we assess convergence using the potential scale reduction factor $\hat{R}$ for each parameter ensuring that $\hat{R}<1.01$ ,^34^ and we check that there are no divergent transitions.^35^ Analysis code and data (including simulated IPD) are available in the online supplementary material, and a tutorial-style walkthrough of the analysis is available as a vignette in the multinma R package.^32^

## Results

### ML-NMR Model

**Figure 2:**
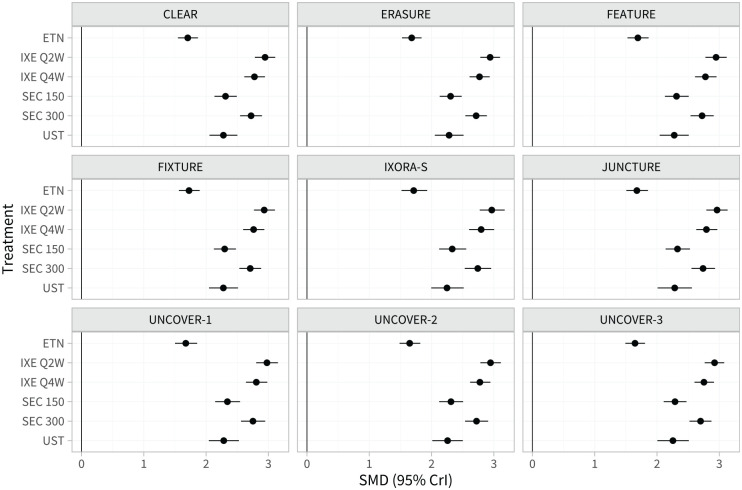
Estimated population-average treatment effects (standardized mean differences) for each treatment versus placebo in the populations represented by each study in the network.

Figure 2 and Table A.4 show the estimated population-average treatment effects for each treatment compared with placebo in each study population. Since the probit link function was used, these can be interpreted as standardized mean differences on the PASI scale. There is little variation in the population-average treatment effects between populations; this is due to the differences in effect modifier distributions between study populations (shown in Table A.2) being small when combined with the estimated strength of the interactions (Table 1).

**Table 1:** Estimated Interactions for Each Treatment Class and Potential Effect Modifier, and Estimated Individual-Level Treatment Effects for an Individual at the Reference/Centering Values of the Covariates (18.2-y Disease Duration, 29.8% Body Surface Area, 89.3 kg Weight, No Previous Systemic Treatment or Psoriatic Arthritis), Using the ML-NMR model^a^

	Treatment Class		
	TNF-α Blocker	IL-17 Blocker	IL-12/23 Blocker
Effect modifier interaction			
Duration of psoriasis, per 10 y	0.17 (0.02, 0.32)	0.17 (0.02, 0.30)	0.12 (−0.08, 0.33)
Previous systemic use	0.11 (−0.27, 0.48)	0.13 (−0.21, 0.46)	−0.01 (−0.69, 0.67)
Body surface area, per 10%	0.04 (−0.06, 0.15)	0.01 (−0.09, 0.11)	0.05 (−0.09, 0.20)
Weight, per 10 kg	−0.09 (−0.17, −0.02)	−0.05 (−0.12, 0.02)	−0.04 (−0.14, 0.07)
Psoriatic arthritis	0.01 (−0.42, 0.49)	0.28 (−0.12, 0.71)	0.32 (−0.33, 1.02)
Reference individual treatment effect			
Etanercept	1.61 (1.35, 1.87)		
Ixekizumab every 2 wk		2.80 (2.55, 3.06)	
Ixekizumab every 4 wk		2.63 (2.38, 2.90)	
Secukinumab 150 mg		2.17 (1.91, 2.43)	
Secukinumab 300 mg		2.58 (2.33, 2.84)	
Ustekinumab			2.21 (1.66, 2.76)

IL, interleukin; ML-NMR, multilevel network meta-regression; TNF, tumor necrosis factor.

a The shared effect modifier assumption was made for ixekizumab and secukinumab, which are both IL-17 blockers, and so these treatments share interaction terms. Etanercept and ustekinumab were treated as belonging to separate treatment classes (TNF-α blocker and IL-12/23 blocker, respectively) and given independent interaction terms. All estimates are standardized mean differences versus placebo, with 95% credible intervals.

**Figure 3:**
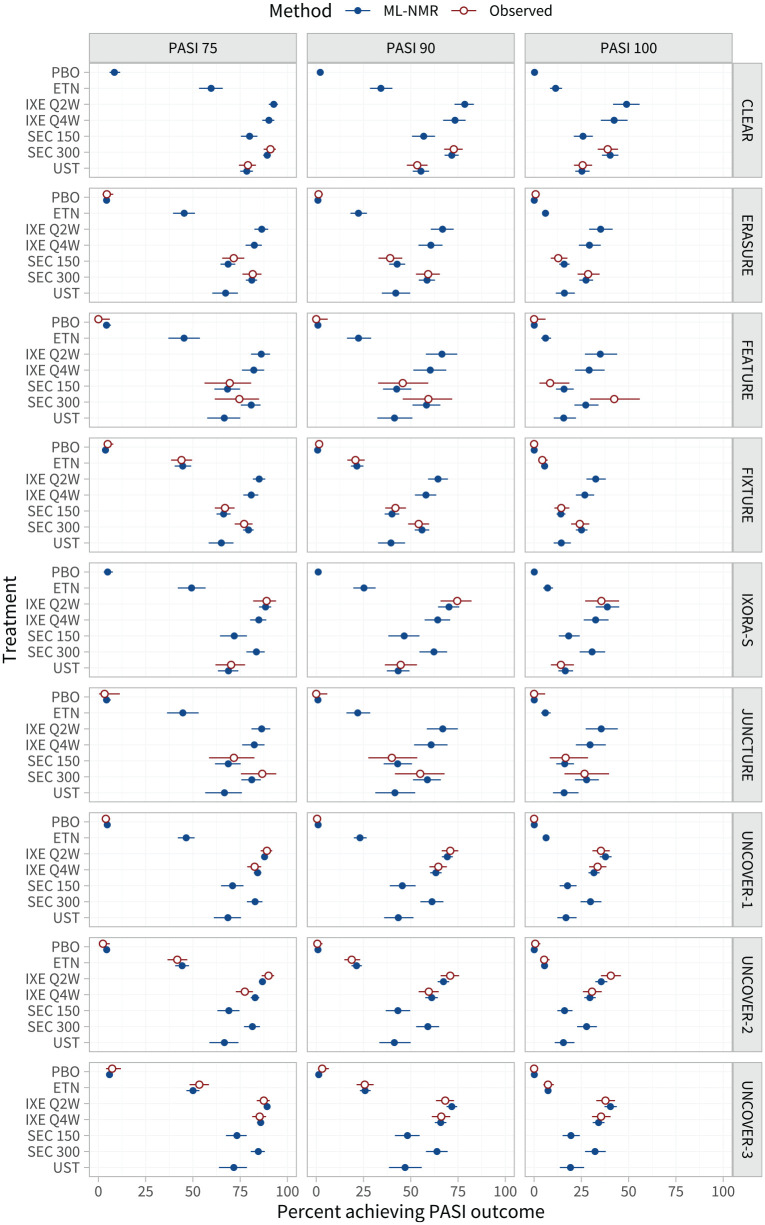
Estimated percentage of individuals achieving each Psoriasis Area and Severity Index (PASI) endpoint on each treatment, in the populations represented by each study in the network. For interpretability, these are given as inclusive probabilities (e.g., the probability of achieving 75% reduction or greater in PASI score). The observed event proportions are calculated from the event counts and sample sizes in each arm.

The estimated proportion of individuals in each study population achieving each PASI outcome are shown in Figure 3 and listed in Tables A.5 to A.7.

We carried out sensitivity analyses, removing covariates from the model (backward selection). The best-fitting model based on the DIC was a model omitting body surface area (DIC 8810.1, residual deviance 8777.1). The estimated population-average treatment effects and event probabilities under this model were very similar to the prespecified analysis model.

### Assessing Residual Heterogeneity and Inconsistency

Checking for residual heterogeneity using a RE ML-NMR model, the estimated heterogeneity standard deviation is 0.09 (0.01, 0.23), which is small compared with the relative treatment effects. Model fit statistics under each model are presented in Table 2. The DIC for the RE ML-NMR model is very similar to the FE ML-NMR model (8815.0 and 8814.9, respectively), and we would choose the more parsimonious FE model based on the DIC alone. Checking for residual inconsistency using a UME ML-NMR model (with FE), this has a DIC of 8817.2, which is also very similar to the FE ML-NMR model assuming consistency. Plotting the residual deviance contributions from the consistency and inconsistency models (Figure A.1) does not suggest that any points fit better under either model. We conclude that there is no evidence for substantial residual heterogeneity or inconsistency after population adjustment: we have detected no failures in the assumptions for the FE ML-NMR model.

**Table 2:** Model Fit Statistics for Each ML-NMR and NMA Model Considered (FE, RE, and UME)^a^

	ML-NMR			NMA		
	FE	RE	UME	FE	RE	UME
Residual deviance	8778.3	8773.2	8779.4	8931.4	8925.0	8932.3
$p_{D}$	36.3	41.8	37.8	16.8	22.4	17.7
DIC	8814.9	8815.0	8817.2	8948.2	8947.5	8950.1
τ	—	0.09 (0.01, 0.23)	—	—	0.09 (0.01, 0.24)	—

ML-NMR, multilevel network meta-regression; NMA, network meta-analysis; FE, fixed effects; RE, random effects; UME, unrelated mean effects.

a $p_{D}$ is a measure of the effective number of parameters. Residual deviance on 12,384 data points. Estimates and 95% credible intervals for the heterogeneity standard deviation τ are also presented for the RE models.

Results from fitting the standard AgD NMA models (Appendix A.4) show little evidence for between-study heterogeneity or inconsistency, yet despite this, the ML-NMR model still has a much lower DIC (Table 2). The ML-NMR model allows us to explain both between- and within-study variation, resulting in better fit and reduced uncertainty in contrast estimates across the study populations (Table A.4).

### Assessing the Shared Effect Modifier Assumption

We assess the shared effect modifier assumption, one covariate at a time, by splitting the common interaction parameter for the covariate in question. Figure A.3 compares the posterior distributions of the independent interactions under each of the split models with the corresponding common interaction from the model making the shared effect modifier assumption for all covariates. In general, the independent interaction estimates are very similar to the common interaction estimates. The only exception is for weight, for which there is some suggestion that this covariate may interact differently with the secukinumab treatment regimens as compared with the ixekizumab regimens. However, the credible intervals for the secukinumab interactions are wide and overlap those for the ixekizumab regimens and the common interaction. In general, all of the independent interaction estimates are much more uncertain for the secukinumab regimens than for the ixekizumab regimens, as the secukinumab parameters are based solely on AgD, and the ixekizumab data largely drive the interaction estimates in the common interaction model. The DIC values (Table A.10) are higher for each of the independent interaction models than for the common interaction model, except for the independent weight interaction model, for which the DIC values are nearly identical. Overall, there is some weak evidence that the shared effect modifier assumption (for the class of interleukin-17 blockers) may be invalid for weight; we consider this further in the discussion. Results from the ML-NMR model with independent weight interactions are given in Appendix A.5 and are very similar to those for the model making the shared effect modifier assumption for all covariates.

### Producing Population-Average Estimates for External Target Populations of Interest

**Figure 4:**
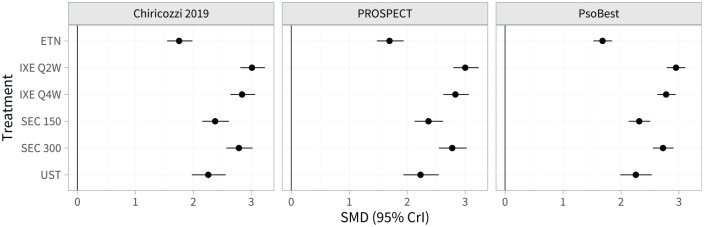
Estimated population-average treatment effects (standardized mean differences) for each treatment versus placebo in each external target population.

**Figure 5:**
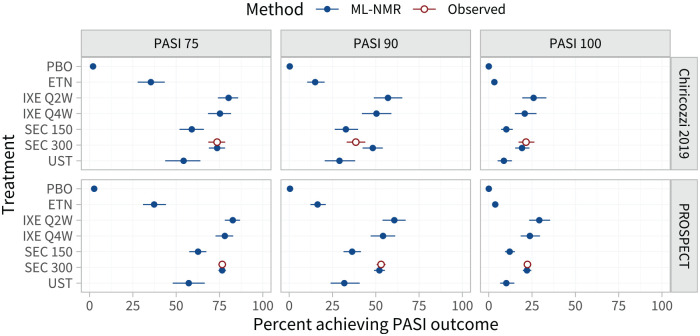
Estimated percentage of individuals achieving each Psoriasis Area and Severity Index (PASI) endpoint on each treatment, in each external target population with information on response rates. For interpretability, these are given as inclusive probabilities (e.g., the probability of achieving 75% reduction or greater in PASI score).

Estimated population-adjusted average treatment effects in the 3 external target populations are shown in Figure 4 and Table A.8. These are similar between the external target populations and the RCTs in the network, again because the differences in effect modifier distributions (Tables A.2 and A.3) are small when combined with the estimated strength of the interactions (Table 1). However, the estimated population-average probabilities of achieving each PASI outcome in the external target populations (Figure 5; Table A.9) are lower than in the RCTs in the network, since the observed proportions achieving PASI 75 on secukinumab 300 mg are lower in the external target populations.

## Discussion

In this article, we have demonstrated an application of ML-NMR to synthesize PASI 75, 90, and 100 ordered categorical outcomes while adjusting for variables considered to be potential effect modifiers, from a network containing a mixture of IPD and AgD studies. The ML-NMR analysis presented here also addresses several issues with previous MAIC analyses of this network.^15^ In particular, ML-NMR makes full use of all available data in one coherent analysis that appropriately quantifies uncertainty (data are not reused), and estimated average treatment effects and average response probabilities for each PASI outcome can be produced in any of the included study populations or in an external target population, whichever is most relevant for decision making. Moreover, this analysis produces more precise estimates than the previous MAIC analyses, and the 95% credible interval for the ixekizumab every 2 weeks versus the secukinumab 300 mg relative effect (the focal comparison of the MAIC analyses) lies further from the null.

The synthesis of the 3 PASI outcomes does require additional assumptions. These are not central to the ML-NMR analyses presented here and are described elsewhere.^4^

All population-adjustment methods, including ML-NMR, assume that all effect modifiers have been suitably adjusted for so that the conditional constancy of relative effects assumption holds.^11^ For pairwise indirect comparisons, including MAIC and STC, this is an untestable assumption. However, in larger networks, failures of conditional constancy of relative effects may manifest as residual heterogeneity or inconsistency, which can be assessed using standard techniques from AgD NMA. In this analysis, we used an RE model and a UME model to check for residual heterogeneity and inconsistency, respectively, and concluded that there was no evidence of residual heterogeneity or inconsistency. However, just as in standard AgD NMA, these techniques may have low power, and the detection of any unobserved effect modifiers relies on these being distributed differently at the aggregate level between studies. Moreover, these methods are fundamentally “in-sample” checks for heterogeneity and inconsistency—there may well be other omitted or unobserved effect modifiers that differ between the studies in the network and an external target population. In a decision-making context, this possibility should be considered when appraising the representativeness of the studies to the decision target population. Although we assessed inconsistency using the UME model, other inconsistency models such as node-splitting^13,21^ or design-by-treatment interactions^36^ may also be implemented within the ML-NMR framework. Currently, the multinma R package implements both UME and node-splitting models for assessing inconsistency.^32^

In this analysis, we relied on the shared effect modifier assumption within the class of interleukin-17 blockers to support the estimation of effect modifier interaction terms. We assessed this assumption, one covariate at a time, by fitting independent interactions for the covariate in question. Exchangeable interactions within each treatment class could also be considered^13^; however, this was not possible given the data available. Relaxing the shared effect modifier assumption for multiple covariates at once is also a possibility, if sufficient data are available. However, for the purposes of checking this assumption, relaxing one covariate at a time should be sufficient to highlight any violations. There was some weak evidence that the shared effect modifier assumption might not be valid for weight, as there were some differences between the independent interaction estimates and the common interaction estimate. However, there was high uncertainty in the independent interaction estimates for the secukinumab regimens, as these were estimated entirely from the 5 AgD studies, and model fit was unchanged. It is likely that this approach to assessing the shared effect modifier assumption has low power, particularly when data are lacking. There is also the possibility that these are chance findings due to fitting multiple models. Nevertheless, the analyses that we propose allow the validity and impact of this assumption to be investigated, even with these caveats, which was not previously possible. In general, differences in estimated interactions may be due to genuinely different interaction effects invalidating the shared effect modifier assumption or due to an imbalance in unobserved effect modifiers across the studies that are correlated with weight. The latter leads to study-level confounding introducing bias into the interaction estimates (“ecological” bias)^37^ and is the reason why meta-analytic studies of effect modification typically split interaction estimates into between-study interaction terms (which are susceptible to study-level confounding) and within-study interaction terms (which are then expected to be unbiased).^38,39^ However, for the purposes of population adjustment, we do not fit a split interaction model, since we must assume that there are no unobserved effect modifiers in order to produce population-adjusted estimates for a target population of interest. Nevertheless, it is possible to fit ML-NMR models where the interaction terms are split in this manner (given sufficient data), and this is an interesting avenue for further research.

When it is used, the shared effect modifier assumption is likely in many cases to be the most challenging to assess, since it is typically used to identify the model when data are insufficient. However, even in cases in which the shared effect modifier assumption is untestable, ML-NMR still retains other notable benefits over MAIC and STC.^13,28^ In particular, unlike MAIC, ML-NMR does not require full overlap between populations for stable estimation and remains unbiased under the usual extrapolation assumptions. Unlike STC, ML-NMR appropriately handles noncollapsible effect measures and does not combine incompatible (marginal and conditional) estimates. Moreover, the assumptions required by ML-NMR are still weaker than those for a standard NMA and indirect comparison, even when they are untestable. In an indirect comparison where the shared effect modifier assumption does not hold, the estimates from ML-NMR will be unbiased and applicable only within the AgD study population, as is the case with MAIC and STC. Small networks with multiple treatment classes and limited IPD are problematic for all current population adjustment methods, as there is likely to be insufficient information to combine the estimates in a coherent manner. In this analysis, even with a relatively small network (9 studies), we had sufficient data to relax and assess the key assumptions, checking for residual heterogeneity and inconsistency and assessing the shared effect modifier assumption.

When fitting network meta-regression models, as well as assuming consistency of the relative treatment effects, there is a similar assumption of consistency on the effect modifier interactions. Donegan et al.^30^ describe methods for assessing potential inconsistency in the effect modifier interactions, analogous to the node-splitting approach^21^ for assessing inconsistency in treatment effects (described for ML-NMR models by Phillippo^14^). However, this network has only 1 potential loop where inconsistency could be present (via ustekinumab) since all other comparisons are made in multiarm trials, and within this loop, the data are insufficient to relax consistency and estimate unrelated interactions.

For the purposes of decision making when effect modification is present, it is crucial that population-adjusted estimates are produced that are specific to the decision target population, whichever population-adjustment method is used.^11^ Ideally, the decision target population is represented by a suitable registry or cohort study^11^; we have produced estimates relevant to the populations represented by registries and a cohort study. Each of these external target populations lay within the range of covariate values in the RCTs; when this is not the case, the validity of extrapolation should be considered. General guidance on when and how to use population-adjustment methods in a health technology assessment context is available.^11^

When decisions are based on cost-effectiveness, the relevant inputs to the economic model are (for a binary or categorical outcome) typically estimated event probabilities on each treatment in the target population. This requires information on the event probability on one treatment in the target population, in addition to the covariate distribution. If this information is not available, expert opinion or event probabilities from a similar population may be used.

When noncollapsible effect measures are used, for example, odds ratios, hazard ratios, or the probit scale used here, ML-NMR typically targets a different population-average treatment effect estimand to MAIC (and indeed to standard AgD NMA based on event counts). The population-average treatment effects typically produced by ML-NMR target a population-average conditional estimand, the same as that targeted by an RCT in the target population using an analysis adjusting for baseline prognostic factors. On the other hand, MAIC (and NMA of event counts) targets a (population-average) marginal estimand, the same as that targeted by an RCT in the target population using an analysis without adjustment for baseline prognostic factors (or better from an adjusted analysis that has been marginalized over the covariates^40^). ML-NMR can also produce estimates of these marginal treatment effects, as we describe in Appendix A.1.2. Importantly, the marginal and conditional estimands have different interpretations when the effect measure is noncollapsible and correspond to different decision questions. The population-average conditional treatment effect is the average effect between randomly selected treated and untreated individuals with the same covariates averaged over the distribution of covariates in the population,^41^ and it answers the decision question, “Which treatment has the greatest effect, on average, in this population?” The marginal population-average treatment effect is the average effect between randomly selected treated and untreated individuals in the population, regardless of their covariates^41^ and answers the decision question, “Which treatment minimizes (or maximizes) the average event probability in this population?” In the absence of effect modification, these decision questions are aligned and always result in the same ranking of treatments, but this is not necessarily the case when effect modification is present, because then different treatments may be more effective for different individuals or subgroups within the population. Moreover, the population-average conditional treatment effects depend only on the distribution of effect-modifying covariates, whereas the marginal treatment effects depend on the distributions of baseline risk, prognostic and effect-modifying covariates, and PASI cutpoint. It is widely understood that when there is patient heterogeneity (including effect modification), health economic analyses need to appropriately handle this by averaging net benefit over the population,^42^ for example, by discrete event simulation.^43,44^ ML-NMR can produce estimates of both the conditional and marginal estimands and necessary quantities for decision models such as average event probabilities or subgroup/individual event probabilities (see Appendix A.1.2).

ML-NMR extends the standard NMA framework to incorporate IPD and AgD studies in networks of any size, adjusting for differences in effect modifiers between studies. It reduces to the gold standard IPD network meta-regression when IPD are available from every study and reduces to standard AgD NMA when no covariates are adjusted for.^13^ Moreover, we have demonstrated how techniques from the NMA literature can be used to assess the underlying assumptions of ML-NMR models. ML-NMR also addresses issues with previous methods such as MAIC, STC, and “plug-in” meta-regression, since it synthesizes networks of any size while avoiding aggregation bias and noncollapsibility bias and can produce estimates of quantities of interest in any chosen target population. The R package multinma facilitates the application of ML-NMR models, making these methods available to a wider range of users.^32^

## Supplemental Material

sj-bbl-6-mdm-10.1177_0272989X221117162 – Supplemental material for Validating the Assumptions of Population Adjustment: Application of Multilevel Network Meta-regression to a Network of Treatments for Plaque Psoriasis Supplemental material, sj-bbl-6-mdm-10.1177_0272989X221117162 for Validating the Assumptions of Population Adjustment: Application of Multilevel Network Meta-regression to a Network of Treatments for Plaque Psoriasis by David M. Phillippo, Sofia Dias, A. E. Ades, Mark Belger, Alan Brnabic, Daniel Saure, Yves Schymura and Nicky J. Welton in Medical Decision Making

sj-bib-5-mdm-10.1177_0272989X221117162 – Supplemental material for Validating the Assumptions of Population Adjustment: Application of Multilevel Network Meta-regression to a Network of Treatments for Plaque Psoriasis Supplemental material, sj-bib-5-mdm-10.1177_0272989X221117162 for Validating the Assumptions of Population Adjustment: Application of Multilevel Network Meta-regression to a Network of Treatments for Plaque Psoriasis by David M. Phillippo, Sofia Dias, A. E. Ades, Mark Belger, Alan Brnabic, Daniel Saure, Yves Schymura and Nicky J. Welton in Medical Decision Making

sj-csv-4-mdm-10.1177_0272989X221117162 – Supplemental material for Validating the Assumptions of Population Adjustment: Application of Multilevel Network Meta-regression to a Network of Treatments for Plaque Psoriasis Supplemental material, sj-csv-4-mdm-10.1177_0272989X221117162 for Validating the Assumptions of Population Adjustment: Application of Multilevel Network Meta-regression to a Network of Treatments for Plaque Psoriasis by David M. Phillippo, Sofia Dias, A. E. Ades, Mark Belger, Alan Brnabic, Daniel Saure, Yves Schymura and Nicky J. Welton in Medical Decision Making

sj-docx-3-mdm-10.1177_0272989X221117162 – Supplemental material for Validating the Assumptions of Population Adjustment: Application of Multilevel Network Meta-regression to a Network of Treatments for Plaque Psoriasis Supplemental material, sj-docx-3-mdm-10.1177_0272989X221117162 for Validating the Assumptions of Population Adjustment: Application of Multilevel Network Meta-regression to a Network of Treatments for Plaque Psoriasis by David M. Phillippo, Sofia Dias, A. E. Ades, Mark Belger, Alan Brnabic, Daniel Saure, Yves Schymura and Nicky J. Welton in Medical Decision Making

sj-pdf-1-mdm-10.1177_0272989X221117162 – Supplemental material for Validating the Assumptions of Population Adjustment: Application of Multilevel Network Meta-regression to a Network of Treatments for Plaque Psoriasis Supplemental material, sj-pdf-1-mdm-10.1177_0272989X221117162 for Validating the Assumptions of Population Adjustment: Application of Multilevel Network Meta-regression to a Network of Treatments for Plaque Psoriasis by David M. Phillippo, Sofia Dias, A. E. Ades, Mark Belger, Alan Brnabic, Daniel Saure, Yves Schymura and Nicky J. Welton in Medical Decision Making

sj-pdf-2-mdm-10.1177_0272989X221117162 – Supplemental material for Validating the Assumptions of Population Adjustment: Application of Multilevel Network Meta-regression to a Network of Treatments for Plaque Psoriasis Supplemental material, sj-pdf-2-mdm-10.1177_0272989X221117162 for Validating the Assumptions of Population Adjustment: Application of Multilevel Network Meta-regression to a Network of Treatments for Plaque Psoriasis by David M. Phillippo, Sofia Dias, A. E. Ades, Mark Belger, Alan Brnabic, Daniel Saure, Yves Schymura and Nicky J. Welton in Medical Decision Making
