# Supplementary material for: Validating the Assumptions of Population Adjustment: Application of Multilevel Network Meta-regression to a Network of Treatments for Plaque Psoriasis
Source: Med Decis Making. 2022 Aug 23;43(1):53–67. doi: 10.1177/0272989X221117162 (PMC9742635; doi:10.1177/0272989X221117162)
Supplement: sj-docx-3-mdm-10.1177_0272989X221117162 – Supplemental material for Validating the Assumptions of Population Adjustment: Application of Multilevel Network Meta-regression to a Network of Treatments for Plaque Psoriasis [file sj-docx-3-mdm-10.1177_0272989X221117162.docx]

###############################################################################

# Treatments for moderate-to-severe plaque psoriasis: analysis of ordered

# categorical outcomes using multilevel network meta-regression (ML-NMR)

#

# David M. Phillippo

###############################################################################

# Setup -------------------------------------------------------------------

library(multinma)

# Set up parallel processing

options(mc.cores = parallel::detectCores())

library(dplyr)

library(tidyr)

library(stringr)

library(readr)

library(forcats)

library(ggplot2)

# Fonts for plots

library(showtext)

font_add_google("Source Sans Pro")

showtext_auto()

library(xtable)

# Create output directories

out_dirs <- c("./figures", "./tables", "./rds")

for (p in out_dirs) {

if (!dir.exists(p)) dir.create(p)

}

# Data preparation --------------------------------------------------------

# The multinma package provides datasets of aggregate data

# (plaque_psoriasis_agd) and simulated individual patient data

# (plaque_psoriasis_ipd) which can be used to recreate the analyses of the paper

# For details on these datasets see `?plaque_psoriasis`

# IPD studies

pso_ipd <- plaque_psoriasis_ipd %>%

mutate(

trtc = str_replace_all(trtc, "_", " "),

# Variable transformations

bsa = bsa / 100,

weight = weight / 10,

durnpso = durnpso / 10,

prevsys = as.numeric(prevsys),

psa = as.numeric(psa),

# Treatment classes

trtclass = case_when(trtn == 1 ~ "Placebo",

trtn %in% c(2, 3, 5, 6) ~ "IL-17 blocker",

trtn == 4 ~ "TNFa blocker",

trtn == 7 ~ "IL-12/23 blocker"),

# Check complete cases for covariates of interest

is_complete = complete.cases(durnpso, prevsys, bsa, weight, psa)

) %>%

arrange(studyc, trtn)

# Only a very small proportion of incomplete rows; we simply remove these

pso_ipd %>%

group_by(studyc) %>%

summarise(sum(!is_complete), mean(!is_complete))

pso_ipd <- filter(pso_ipd, is_complete)

# AgD studies

pso_agd <- plaque_psoriasis_agd %>%

mutate(

trtc = str_replace_all(trtc, "_", " "),

# Variable transformations

bsa_mean = bsa_mean / 100,

bsa_sd = bsa_sd / 100,

weight_mean = weight_mean / 10,

weight_sd = weight_sd / 10,

durnpso_mean = durnpso_mean / 10,

durnpso_sd = durnpso_sd / 10,

prevsys = prevsys / 100,

psa = psa / 100,

# Treatment classes

trtclass = case_when(trtn == 1 ~ "Placebo",

trtn %in% c(2, 3, 5, 6) ~ "IL-17 blocker",

trtn == 4 ~ "TNFa blocker",

trtn == 7 ~ "IL-12/23 blocker")

) %>%

arrange(studyc, trtn)

# Create network ----------------------------------------------------------

pso_net <- combine_network(

set_ipd(pso_ipd,

study = studyc,

trt = trtc,

r = multi(r0 = 1,

PASI75 = pasi75,

PASI90 = pasi90,

PASI100 = pasi100,

type = "ordered", inclusive = TRUE),

trt_class = trtclass),

set_agd_arm(pso_agd,

study = studyc,

trt = trtc,

r = multi(r0 = pasi75_n,

PASI75 = pasi75_r,

PASI90 = pasi90_r,

PASI100 = pasi100_r,

type = "ordered", inclusive = TRUE),

trt_class = trtclass)

)

pso_net

# Network plot

plot(pso_net, weight_nodes = TRUE, weight_edges = TRUE, show_trt_class = TRUE)

# Customise for paper

class_pal <- RColorBrewer::brewer.pal(8, "Dark2")[c(2, 3, 4, 6)]

plot(pso_net, weight_nodes = TRUE, weight_edges = TRUE, show_trt_class = TRUE) +

ggraph::scale_edge_colour_manual("Data", values = c(AgD = "#113259", IPD = "#55A480"),

guide = guide_legend(override.aes = list(edge_width = 2))) +

scale_fill_manual("Treatment class",

values = class_pal,

aesthetics = c("fill", "colour"),

guide = guide_legend(override.aes = list(size = 2)))

ggsave("./figures/full_network_plot_paper.pdf", height = 6, width = 7.5)

# Set up numerical integration --------------------------------------------

# Since the full joint distribution is unknown in each AgD study, we assume that

# the marginal forms and correlations match those in the IPD studies

# Here we plot the assumed distributions of the continuous covariates in IPD

# Get mean and sd of covariates in each study

ipd_summary <- pso_ipd %>%

group_by(studyc) %>%

summarise_at(vars(weight, durnpso, bsa), list(mean = mean, sd = sd, min = min, max = max)) %>%

pivot_longer(weight_mean:bsa_max, names_sep = "_", names_to = c("covariate", ".value")) %>%

# Assign distributions

mutate(dist = recode(covariate,

bsa = "dlogitnorm",

durnpso = "dgamma",

weight = "dgamma")) %>%

# Compute density curves

group_by(studyc, covariate) %>%

mutate(value = if_else(dist == "dlogitnorm",

list(seq(0, 1, length.out = 101)),

list(seq(min*0.8, max*1.2, length.out = 101)))) %>%

unnest(cols = value) %>%

mutate(dens = do.call(first(dist), args = list(x = value, mean = first(mean), sd = first(sd))))

# Plot histograms and assumed densities

pso_ipd %>%

pivot_longer(c(weight, durnpso, bsa), names_to = "covariate", values_to = "value") %>%

ggplot(aes(x = value)) +

geom_histogram(aes(y = stat(density)),

binwidth = function(x) diff(range(x)) / nclass.Sturges(x),

boundary = 0,

fill = "grey50") +

geom_line(aes(y = dens), data = ipd_summary,

colour = "darkred", size = 0.5) +

facet_wrap(~studyc + covariate, scales = "free", ncol = 3) +

theme_multinma()

# Add numerical integration points to the network

# By default, correlations are taken as a weighted average of those in the IPD studies

pso_net <- add_integration(pso_net,

durnpso = distr(qgamma, mean = durnpso_mean, sd = durnpso_sd),

prevsys = distr(qbern, prob = prevsys),

bsa = distr(qlogitnorm, mean = bsa_mean, sd = bsa_sd),

weight = distr(qgamma, mean = weight_mean, sd = weight_sd),

psa = distr(qbern, prob = psa),

n_int = 1000)

# Model fitting -----------------------------------------------------------

## FE ML-NMR --------------------------------------------------------------

pso_FE_save <- "./rds/multi_pso_fit_FE.rds"

if (interactive() && file.exists(pso_FE_save) && toupper(readline("Read in saved model fit? [y/n]: ")) == "Y") {

pso_fit_FE <- readRDS(pso_FE_save)

rstan::check_hmc_diagnostics(as.stanfit(pso_fit_FE))

} else {

pso_fit_FE <- nma(pso_net,

trt_effects = "fixed",

link = "probit",

regression = ~(durnpso + prevsys + bsa + weight + psa)*.trt,

class_interactions = "common",

prior_intercept = normal(scale = 10),

prior_trt = normal(scale = 10),

prior_reg = normal(scale = 10),

prior_aux = flat(),

QR = TRUE,

init_r = 0.5,

save_warmup = FALSE)

saveRDS(pso_fit_FE, file = pso_FE_save)

}

pso_fit_FE

plot_prior_posterior(pso_fit_FE)

## RE ML-NMR --------------------------------------------------------------

# Check for residual heterogeneity with a RE model

pso_RE_save <- "./rds/multi_pso_fit_RE.rds"

if (interactive() && file.exists(pso_RE_save) && toupper(readline("Read in saved model fit? [y/n]: ")) == "Y") {

pso_fit_RE <- readRDS(pso_RE_save)

rstan::check_hmc_diagnostics(as.stanfit(pso_fit_RE))

} else {

pso_fit_RE <- nma(pso_net,

trt_effects = "random",

link = "probit",

regression = ~(durnpso + prevsys + bsa + weight + psa)*.trt,

class_interactions = "common",

prior_intercept = normal(scale = 10),

prior_trt = normal(scale = 10),

prior_reg = normal(scale = 10),

prior_aux = flat(),

prior_het = half_normal(scale = 2.5),

QR = TRUE,

init_r = 0.5,

save_warmup = FALSE)

saveRDS(pso_fit_RE, file = pso_RE_save)

}

pso_fit_RE

plot_prior_posterior(pso_fit_RE)

# Compare model fit

(pso_dic_FE <- dic(pso_fit_FE))

(pso_dic_RE <- dic(pso_fit_RE))

# FE ML-NMR relaxing shared EM assumption ---------------------------------

# Fit independent interactions for each covariate one-by-one

pso_FE_SEM_durnpso_save <- "./rds/multi_pso_fit_FE_SEM_durnpso.rds"

if (interactive() && file.exists(pso_FE_SEM_durnpso_save) && toupper(readline("Read in saved model fit? [y/n]: ")) == "Y") {

pso_fit_FE_SEM_durnpso <- readRDS(pso_FE_SEM_durnpso_save)

rstan::check_hmc_diagnostics(as.stanfit(pso_fit_FE_SEM_durnpso))

} else {

pso_fit_FE_SEM_durnpso <- nma(pso_net,

trt_effects = "fixed",

link = "probit",

# Specify common interactions directly by using .trtclass special,

# except for durnpso which is independent interactions with .trt

regression = ~durnpso + prevsys + bsa + weight + psa +

(prevsys + bsa + weight + psa):.trtclass + durnpso:.trt,

# class_interactions only affects formula interaction terms with .trt,

# and we want these to be independent

class_interactions = "independent",

prior_intercept = normal(scale = 10),

prior_trt = normal(scale = 10),

prior_reg = normal(scale = 10),

prior_aux = flat(),

QR = TRUE,

init_r = 0.5,

save_warmup = FALSE)

saveRDS(pso_fit_FE_SEM_durnpso, file = pso_FE_SEM_durnpso_save)

}

pso_fit_FE_SEM_durnpso

pso_FE_SEM_prevsys_save <- "./rds/multi_pso_fit_FE_SEM_prevsys.rds"

if (interactive() && file.exists(pso_FE_SEM_prevsys_save) && toupper(readline("Read in saved model fit? [y/n]: ")) == "Y") {

pso_fit_FE_SEM_prevsys <- readRDS(pso_FE_SEM_prevsys_save)

rstan::check_hmc_diagnostics(as.stanfit(pso_fit_FE_SEM_prevsys))

} else {

pso_fit_FE_SEM_prevsys <- nma(pso_net,

trt_effects = "fixed",

link = "probit",

# Specify common interactions directly by using .trtclass special,

# except for prevsys which is independent interactions with .trt

regression = ~durnpso + prevsys + bsa + weight + psa +

(durnpso + bsa + weight + psa):.trtclass + prevsys:.trt,

# class_interactions only affects formula interaction terms with .trt,

# and we want these to be independent

class_interactions = "independent",

prior_intercept = normal(scale = 10),

prior_trt = normal(scale = 10),

prior_reg = normal(scale = 10),

prior_aux = flat(),

QR = TRUE,

init_r = 0.5,

save_warmup = FALSE)

saveRDS(pso_fit_FE_SEM_prevsys, file = pso_FE_SEM_prevsys_save)

}

pso_fit_FE_SEM_prevsys

pso_FE_SEM_bsa_save <- "./rds/multi_pso_fit_FE_SEM_bsa.rds"

if (interactive() && file.exists(pso_FE_SEM_bsa_save) && toupper(readline("Read in saved model fit? [y/n]: ")) == "Y") {

pso_fit_FE_SEM_bsa <- readRDS(pso_FE_SEM_bsa_save)

rstan::check_hmc_diagnostics(as.stanfit(pso_fit_FE_SEM_bsa))

} else {

pso_fit_FE_SEM_bsa <- nma(pso_net,

trt_effects = "fixed",

link = "probit",

# Specify common interactions directly by using .trtclass special,

# except for bsa which is independent interactions with .trt

regression = ~durnpso + prevsys + bsa + weight + psa +

(durnpso + prevsys + weight + psa):.trtclass + bsa:.trt,

# class_interactions only affects formula interaction terms with .trt,

# and we want these to be independent

class_interactions = "independent",

prior_intercept = normal(scale = 10),

prior_trt = normal(scale = 10),

prior_reg = normal(scale = 10),

prior_aux = flat(),

QR = TRUE,

init_r = 0.5,

save_warmup = FALSE)

saveRDS(pso_fit_FE_SEM_bsa, file = pso_FE_SEM_bsa_save)

}

pso_fit_FE_SEM_bsa

pso_FE_SEM_weight_save <- "./rds/multi_pso_fit_FE_SEM_weight.rds"

if (interactive() && file.exists(pso_FE_SEM_weight_save) && toupper(readline("Read in saved model fit? [y/n]: ")) == "Y") {

pso_fit_FE_SEM_weight <- readRDS(pso_FE_SEM_weight_save)

rstan::check_hmc_diagnostics(as.stanfit(pso_fit_FE_SEM_weight))

} else {

pso_fit_FE_SEM_weight <- nma(pso_net,

trt_effects = "fixed",

link = "probit",

# Specify common interactions directly by using .trtclass special,

# except for weight which is independent interactions with .trt

regression = ~durnpso + prevsys + bsa + weight + psa +

(durnpso + prevsys + bsa + psa):.trtclass + weight:.trt,

# class_interactions only affects formula interaction terms with .trt,

# and we want these to be independent

class_interactions = "independent",

prior_intercept = normal(scale = 10),

prior_trt = normal(scale = 10),

prior_reg = normal(scale = 10),

prior_aux = flat(),

QR = TRUE,

init_r = 0.5,

save_warmup = FALSE)

saveRDS(pso_fit_FE_SEM_weight, file = pso_FE_SEM_weight_save)

}

pso_fit_FE_SEM_weight

pso_RE_SEM_weight_save <- "./rds/multi_pso_fit_RE_SEM_weight.rds"

if (interactive() && file.exists(pso_RE_SEM_weight_save) && toupper(readline("Read in saved model fit? [y/n]: ")) == "Y") {

pso_fit_RE_SEM_weight <- readRDS(pso_RE_SEM_weight_save)

rstan::check_hmc_diagnostics(as.stanfit(pso_fit_RE_SEM_weight))

} else {

pso_fit_RE_SEM_weight <- nma(pso_net,

trt_effects = "random",

link = "probit",

# Specify common interactions directly by using .trtclass special,

# except for weight which is independent interactions with .trt

regression = ~durnpso + prevsys + bsa + weight + psa +

(durnpso + prevsys + bsa + psa):.trtclass + weight:.trt,

# class_interactions only affects formula interaction terms with .trt,

# and we want these to be independent

class_interactions = "independent",

prior_intercept = normal(scale = 10),

prior_trt = normal(scale = 10),

prior_reg = normal(scale = 10),

prior_aux = flat(),

prior_het = half_normal(scale = 2.5),

QR = TRUE,

init_r = 0.5,

save_warmup = FALSE)

saveRDS(pso_fit_RE_SEM_weight, file = pso_RE_SEM_weight_save)

}

pso_fit_RE_SEM_weight

pso_FE_SEM_psa_save <- "./rds/multi_pso_fit_FE_SEM_psa.rds"

if (interactive() && file.exists(pso_FE_SEM_psa_save) && toupper(readline("Read in saved model fit? [y/n]: ")) == "Y") {

pso_fit_FE_SEM_psa <- readRDS(pso_FE_SEM_psa_save)

rstan::check_hmc_diagnostics(as.stanfit(pso_fit_FE_SEM_psa))

} else {

pso_fit_FE_SEM_psa <- nma(pso_net,

trt_effects = "fixed",

link = "probit",

# Specify common interactions directly by using .trtclass special,

# except for psa which is independent interactions with .trt

regression = ~durnpso + prevsys + bsa + weight + psa +

(durnpso + prevsys + bsa + weight):.trtclass + psa:.trt,

# class_interactions only affects formula interaction terms with .trt,

# and we want these to be independent

class_interactions = "independent",

prior_intercept = normal(scale = 10),

prior_trt = normal(scale = 10),

prior_reg = normal(scale = 10),

prior_aux = flat(),

QR = TRUE,

init_r = 0.5,

save_warmup = FALSE)

saveRDS(pso_fit_FE_SEM_psa, file = pso_FE_SEM_psa_save)

}

pso_fit_FE_SEM_psa

# Compare model fit

pso_dic_FE

(pso_dic_FE_SEM_durnpso <- dic(pso_fit_FE_SEM_durnpso))

(pso_dic_FE_SEM_prevsys <- dic(pso_fit_FE_SEM_prevsys))

(pso_dic_FE_SEM_bsa <- dic(pso_fit_FE_SEM_bsa))

(pso_dic_FE_SEM_weight <- dic(pso_fit_FE_SEM_weight))

(pso_dic_FE_SEM_psa <- dic(pso_fit_FE_SEM_psa))

# Plot of covariate interaction terms under each model

bind_rows(

as_tibble(as.matrix(pso_fit_FE, pars = "beta")) %>%

pivot_longer(cols = everything(), names_to = "parameter", values_to = "value") %>%

mutate(model = "all",

value = if_else(str_detect(parameter, "bsa"), value / 10, value)),

as_tibble(as.matrix(pso_fit_FE_SEM_durnpso, pars = "beta")) %>%

pivot_longer(cols = everything(), names_to = "parameter", values_to = "value") %>%

mutate(model = "durnpso"),

as_tibble(as.matrix(pso_fit_FE_SEM_prevsys, pars = "beta")) %>%

pivot_longer(cols = everything(), names_to = "parameter", values_to = "value") %>%

mutate(model = "prevsys"),

as_tibble(as.matrix(pso_fit_FE_SEM_bsa, pars = "beta") / 10) %>%

pivot_longer(cols = everything(), names_to = "parameter", values_to = "value") %>%

mutate(model = "bsa"),

as_tibble(as.matrix(pso_fit_FE_SEM_weight, pars = "beta")) %>%

pivot_longer(cols = everything(), names_to = "parameter", values_to = "value") %>%

mutate(model = "weight"),

as_tibble(as.matrix(pso_fit_FE_SEM_psa, pars = "beta")) %>%

pivot_longer(cols = everything(), names_to = "parameter", values_to = "value") %>%

mutate(model = "psa"),

) %>%

filter((model == "all" & str_detect(parameter, ":.+IL\\-17 blocker")) |

(model == "durnpso" & str_detect(parameter, "(IXE|SEC).+:durnpso")) |

(model == "prevsys" & str_detect(parameter, "(IXE|SEC).+:prevsys")) |

(model == "bsa" & str_detect(parameter, "(IXE|SEC).+:bsa")) |

(model == "weight" & str_detect(parameter, "(IXE|SEC).+:weight")) |

(model == "psa" & str_detect(parameter, "(IXE|SEC).+:psa"))) %>%

mutate(covariate = str_extract(parameter, "durnpso|prevsys|bsa|weight|psa"),

covariatef = recode_factor(covariate,

durnpso = "Duration of psoriasis, per 10 years",

prevsys = "Previous systemic use",

bsa = "Body surface area, per 10%",

weight = "Weight, per 10 kg",

psa = "Psoriatic arthritis"),

treatment = str_remove(str_extract(parameter, "\\.trt(class)?.+?(?=[\\]:])"), "\\.trt(class)?"),

Interactions = fct_collapse(factor(model),

Common = "all",

other_level = "Independent")) %>%

# Plot

ggplot(aes(x = value, y = fct_rev(treatment), colour = Interactions, fill = Interactions)) +

geom_vline(xintercept = 0, colour = "grey70") +

ggdist::stat_halfeye(normalize = "panels", slab_alpha = 0.3, .width = c(0, 0.95)) +

facet_wrap("covariatef", scales = "free") +

xlab("Interaction effect (SMD)") +

ylab("Treatment / Class") +

scale_colour_manual(values = c(Common = "#7B3294", Independent = "#91D388"),

aesthetics = c("colour", "fill")) +

theme_multinma(base_family = "Source Sans Pro") +

theme(legend.position = c(0.85, 0.2))

ggsave("./figures/pso_multi_interaction_check.pdf", width = 8, height = 5)

## UME ML-NMR -------------------------------------------------------------

# Check for residual inconsistency with an unrelated mean effects model

pso_UME_save <- "./rds/multi_pso_fit_UME.rds"

if (interactive() && file.exists(pso_UME_save) && toupper(readline("Read in saved model fit? [y/n]: ")) == "Y") {

pso_fit_UME <- readRDS(pso_UME_save)

rstan::check_hmc_diagnostics(as.stanfit(pso_fit_UME))

} else {

pso_fit_UME <- nma(pso_net,

trt_effects = "fixed",

consistency = "ume",

link = "probit",

regression = ~(durnpso + prevsys + bsa + weight + psa)*.trt,

class_interactions = "common",

prior_intercept = normal(scale = 10),

prior_trt = normal(scale = 10),

prior_reg = normal(scale = 10),

prior_aux = flat(),

QR = TRUE,

init_r = 0.5,

save_warmup = FALSE)

saveRDS(pso_fit_UME, file = pso_UME_save)

}

pso_fit_UME

plot_prior_posterior(pso_fit_UME)

# Compare model fit

pso_dic_FE

(pso_dic_UME <- dic(pso_fit_UME))

# dev-dev plot of residual deviance contributions

plot(pso_dic_FE, pso_dic_UME, show_uncertainty = FALSE)

# Customise for paper

plot(pso_dic_FE, pso_dic_UME,

.width = c(0, 0.95),

point_alpha = 0.75, interval_alpha = 0.25) +

xlab("Residual deviance - consistency model") +

ylab("Residual deviance - inconsistency (UME) model") +

scale_colour_manual("", values = c(IPD = "#ABDDA4", "AgD (arm-based)" = "#7B3294")) +

aes(shape = Type) +

scale_shape_manual("", values = c(IPD = 16, "AgD (arm-based)" = 15)) +

theme(legend.position = "top", legend.direction = "horizontal",

legend.margin = margin(0, 0, 0, 0))

ggsave("./figures/multi_dev_dev_FEMLNMR_UMEMLNMR.pdf", width = 5, height = 5.2, scale = 0.9)

## FE NMA -----------------------------------------------------------------

# Compare ML-NMR with unadjusted NMA models

pso_FE_NMA_save <- "./rds/multi_pso_fit_FE_NMA.rds"

if (interactive() && file.exists(pso_FE_NMA_save) && toupper(readline("Read in saved model fit? [y/n]: ")) == "Y") {

pso_fit_FE_NMA <- readRDS(pso_FE_NMA_save)

rstan::check_hmc_diagnostics(as.stanfit(pso_fit_FE_NMA))

} else {

pso_fit_FE_NMA <- nma(pso_net,

trt_effects = "fixed",

link = "probit",

prior_intercept = normal(scale = 10),

prior_trt = normal(scale = 10),

prior_aux = flat(),

save_warmup = FALSE)

saveRDS(pso_fit_FE_NMA, file = pso_FE_NMA_save)

}

pso_fit_FE_NMA

plot_prior_posterior(pso_fit_FE_NMA)

## RE NMA -----------------------------------------------------------------

pso_RE_NMA_save <- "./rds/multi_pso_fit_RE_NMA.rds"

if (interactive() && file.exists(pso_RE_NMA_save) && toupper(readline("Read in saved model fit? [y/n]: ")) == "Y") {

pso_fit_RE_NMA <- readRDS(pso_RE_NMA_save)

rstan::check_hmc_diagnostics(as.stanfit(pso_fit_RE_NMA))

} else {

pso_fit_RE_NMA <- nma(pso_net,

trt_effects = "random",

link = "probit",

prior_intercept = normal(scale = 10),

prior_trt = normal(scale = 10),

prior_aux = flat(),

prior_het = half_normal(scale = 2.5),

save_warmup = FALSE)

saveRDS(pso_fit_RE_NMA, file = pso_RE_NMA_save)

}

pso_fit_RE_NMA

plot_prior_posterior(pso_fit_RE_NMA)

# Compare model fit

(pso_dic_FE_NMA <- dic(pso_fit_FE_NMA))

(pso_dic_RE_NMA <- dic(pso_fit_RE_NMA))

## UME NMA ----------------------------------------------------------------

pso_UME_NMA_save <- "./rds/multi_pso_fit_UME_NMA.rds"

if (interactive() && file.exists(pso_UME_NMA_save) && toupper(readline("Read in saved model fit? [y/n]: ")) == "Y") {

pso_fit_UME_NMA <- readRDS(pso_UME_NMA_save)

rstan::check_hmc_diagnostics(as.stanfit(pso_fit_UME_NMA))

} else {

pso_fit_UME_NMA <- nma(pso_net,

trt_effects = "fixed",

consistency = "ume",

link = "probit",

prior_intercept = normal(scale = 10),

prior_trt = normal(scale = 10),

prior_aux = flat(),

save_warmup = FALSE)

saveRDS(pso_fit_UME_NMA, file = pso_UME_NMA_save)

}

pso_fit_UME_NMA

plot_prior_posterior(pso_fit_UME_NMA)

# Compare model fit

pso_dic_FE_NMA

(pso_dic_UME_NMA <- dic(pso_fit_UME_NMA))

# dev-dev plot of residual deviance contributions

plot(pso_dic_FE_NMA, pso_dic_UME_NMA, show_uncertainty = FALSE)

# Customise for paper

plot(pso_dic_FE_NMA, pso_dic_UME_NMA,

.width = c(0, 0.95),

point_alpha = 0.75, interval_alpha = 0.25) +

xlab("Residual deviance - consistency model") +

ylab("Residual deviance - inconsistency (UME) model") +

scale_colour_manual("", values = c(IPD = "#ABDDA4", "AgD (arm-based)" = "#7B3294")) +

aes(shape = Type) +

scale_shape_manual("", values = c(IPD = 16, "AgD (arm-based)" = 15)) +

theme(legend.position = "top", legend.direction = "horizontal",

legend.margin = margin(0, 0, 0, 0))

ggsave("./figures/multi_dev_dev_FENMA_UMENMA.pdf", width = 5, height = 5.2, scale = 0.9)

# Results -----------------------------------------------------------------

# Relative effects

(pso_releff_FE <- relative_effects(pso_fit_FE))

plot(pso_releff_FE, ref_line = 0) +

facet_wrap("Study")

# Predicted probabilities

pso_pred_FE_save <- "./rds/multi_pso_pred_FE.rds"

if (interactive() && file.exists(pso_pred_FE_save) && toupper(readline("Read in saved results? [y/n]: ")) == "Y") {

pso_pred_FE <- readRDS(pso_pred_FE_save)

} else {

pso_pred_FE <- predict(pso_fit_FE, type = "response")

saveRDS(pso_pred_FE, pso_pred_FE_save)

}

pso_pred_FE

plot(pso_pred_FE)

## New target populations -------------------------------------------------

# Covariate means and SDs in target populations

new_agd_means <- read_csv("./registry_summaries.csv") %>%

pivot_wider(id_cols = study,

names_from = covariate,

values_from = c(mean, sd),

names_glue = "{covariate}_{.value}") %>%

transmute(study,

age_mean, age_sd,

bsa_mean, bsa_sd,

durnpso_mean, durnpso_sd,

pasi_w0_mean, pasi_w0_sd,

prevsys = prevsys_mean,

psa = psa_mean,

male = male_mean,

weight_mean, weight_sd)

# Rescale as per analysis

new_agd_means_rs <- transmute(new_agd_means,

study,

bsa_mean = bsa_mean / 100,

bsa_sd = bsa_sd / 100,

weight_mean = weight_mean / 10,

weight_sd = weight_sd / 10,

durnpso_mean = durnpso_mean / 10,

durnpso_sd = durnpso_sd / 10,

prevsys = prevsys,

psa = psa

)

# Relative effects

(pso_releff_FE_new <- relative_effects(pso_fit_FE,

newdata = transmute(new_agd_means_rs,

study,

bsa = bsa_mean,

weight = weight_mean,

durnpso = durnpso_mean,

prevsys,

psa),

study = study))

plot(pso_releff_FE_new, ref_line = 0) + facet_wrap("Study")

# Absolute response probabilities

new_agd_int <- add_integration(filter(new_agd_means_rs, study != "PsoBest"),

durnpso = distr(qgamma, mean = durnpso_mean, sd = durnpso_sd),

prevsys = distr(qbern, prob = prevsys),

bsa = distr(qlogitnorm, mean = bsa_mean, sd = bsa_sd),

weight = distr(qgamma, mean = weight_mean, sd = weight_sd),

psa = distr(qbern, prob = psa),

n_int = 1000,

cor = pso_net$int_cor)

(pso_pred_FE_new <- predict(pso_fit_FE,

type = "response",

newdata = new_agd_int,

study = study,

baseline = list(PROSPECT = distr(qbeta, 1156, 1509-1156),

"Chiricozzi 2019" = distr(qbeta, 243, 330-243)),

baseline_type = "response",

baseline_level = "aggregate",

trt_ref = "SEC 300"))

plot(pso_pred_FE_new, ref_line = c(0, 1)) +

facet_wrap("Study") +

aes(colour = Category) +

scale_colour_brewer(palette = "Blues")

# Outputs for paper -------------------------------------------------------

n_X <- 5 # Number of covariates

ntrt <- 7 # Number of treatments

# Function for formatting tables

tabfmt <- function(x, digits = 2, format = "f", flag = "#",

trunc.lo = 0,

na.char = "-",

prefix = "$", suffix = "$", ...) {

if (length(digits) > 1) {

out <- mapply(formatC, x, digits, MoreArgs = list(format = format, flag = flag, ...))

} else {

out <- formatC(x, digits = digits, format = format, flag = flag, ...)

}

out[x > 0 & x < trunc.lo] <- paste0("<", trunc.lo)

out[x < 0 & x > trunc.lo] <- paste0(">-", trunc.lo)

out[is.na(x) | is.nan(x)] <- na.char

if (!is.null(prefix) || !is.null(suffix)) out <- paste0(prefix, out, suffix)

return(out)

}

## Table of parameter estimates -------------------------------------------

# Basic parameter estimates

summary(pso_fit_FE, pars = c("beta", "d"))

# Report individual-level treatment effect at base level of factors

pasi_multi_estimates <- bind_rows(

as_tibble(summary(pso_fit_FE, pars = "beta")),

as_tibble(relative_effects(pso_fit_FE,

newdata = tibble(!!! pso_fit_FE$xbar) %>%

mutate(prevsys = 0,

psa = 0)))

) %>%

select(-.study)

print(pasi_multi_estimates, n = Inf)

pasi_multi_estimates %>%

as_tibble() %>%

mutate(par_base = gsub("^(.*)\\[.+\\]$", "\\1", parameter)) %>%

# Remove parameters for prognostic terms (only EMs and trt effects)

filter(!grepl("^beta\\[[^:]+\\]$", parameter)) %>%

mutate(trtclass_raw = if_else(par_base == "beta",

gsub(".+:\\.trtclass(.+)\\]", "\\1", parameter),

gsub("^d\\[New 1: (.+)\\]$", "\\1", parameter)),

trtclass = factor(recode(trtclass_raw,

"IXE Q2W" = "IL-17 blocker",

"IXE Q4W" = "IL-17 blocker",

ETN = "TNFa blocker",

"SEC 150" = "IL-17 blocker",

"SEC 300" = "IL-17 blocker",

UST = "IL-12/23 blocker"),

levels = levels(pso_net$classes)),

xvar = if_else(par_base == "beta",

gsub("^beta\\[(.+):\\.trtclass.+", "\\1", parameter),

gsub("^d\\[New 1: (.+)\\]$", "\\1", parameter)),

Covariate = recode(xvar,

prevsys = "Previous systemic use",

durnpso = "Duration of psoriasis, per 10 years",

bsa = "Body surface area, per 10\\%",

weight = "Weight, per 10 kg",

psa = "Psoriatic arthritis"),

# Set digits for output

dg = recode(par_base,

beta = 2, d = 2),

# Rescale body surface area to per 10%

mean = if_else(xvar == "bsa", mean/10, mean),

`2.5%` = if_else(xvar == "bsa", `2.5%`/10, `2.5%`),

`97.5%` = if_else(xvar == "bsa", `97.5%`/10, `97.5%`),

est = paste0(tabfmt(mean, dg),

" (", tabfmt(`2.5%`, dg), ", ", tabfmt(`97.5%`, dg), ")")) %>%

mutate(row_id = rep(1:(n_X + ntrt-1), rle(xvar)$lengths)) %>%

select(row_id, Covariate, est, trtclass) %>%

spread(trtclass, est) %>%

select(-row_id) %>%

# Add block headers

add_row(Covariate = "\\rowgroup{Effect modifier interaction}", .before = 1) %>%

add_row(Covariate = "\\rowgroup{Reference individual treatment effect}", .after = n_X + 1) %>%

tibble::column_to_rownames("Covariate") %>%

# Output to latex

xtable() %>%

print(file = "./tables/pso_multi_interactions.tex",

include.rownames = TRUE,

sanitize.text.function = function(x) {x},

sanitize.colnames.function = function(x) {x},

sanitize.rownames.function = function(x) {x},

hline.after = c(-1, 0, n_X + 1, n_X + ntrt + 1),

booktabs = TRUE,

only.contents = TRUE)

## Table of population-average relative effects ---------------------------

relative_effects(pso_fit_FE, all_contrasts = TRUE) %>%

as_tibble() %>%

# Add in estimates from FE NMA

bind_rows(

relative_effects(pso_fit_FE_NMA, all_contrasts = TRUE) %>%

as_tibble() %>%

mutate(.study = "FE NMA")

) %>%

mutate(trtb = factor(gsub("^d\\[(.+: )?(.+) vs\\. (.+)\\]$", "\\2", parameter),

levels = levels(pso_net$treatments)),

trta = factor(gsub("^d\\[(.+: )?(.+) vs\\. (.+)\\]$", "\\3", parameter),

levels = levels(pso_net$treatments))) %>%

# Take comparisons against placebo, selected focal comparisons

filter(trta == "PBO" |

(trta %in% c("IXE Q2W", "SEC 300", "UST") &

trtb %in% c("IXE Q2W", "SEC 300", "UST"))) %>%

# Format estimates and CrIs

transmute(trtb, trta,

`Study population` = .study,

Contrast = paste0(trtb, " vs.\\ ", trta),

interval = if_else(is.na(mean), "",

paste0("(", tabfmt(`2.5%`), ", ",

tabfmt(`97.5%`), ")")

),

est = if_else(is.na(mean), "-", tabfmt(mean))

) %>%

# Transpose into table

gather(stat, value, est, interval) %>%

pivot_wider(names_from = `Study population`, values_from = value) %>%

arrange(trta, trtb, stat) %>%

group_by(Contrast) %>% mutate(grpid = 1:n()) %>% ungroup() %>%

mutate(Contrast = if_else(grpid == 1, Contrast, "")) %>%

select(-stat, -trta, -trtb, -grpid) %>%

# Output to Latex

xtable() %>%

print(file = "./tables/pso_multi_contrasts.tex",

sanitize.colnames.function = function(x) {x},

sanitize.text.function = function(x) {x},

add.to.row = list(pos = as.list(seq(2, nrow(.), 2)), command = rep("[1.5ex]", nrow(.)/2)),

include.rownames = FALSE,

booktabs = TRUE,

only.contents = TRUE,

comment = FALSE)

## Table of population-average relative effects (target pops) -------------

relative_effects(pso_fit_FE,

newdata = transmute(new_agd_means_rs,

study,

bsa = bsa_mean,

weight = weight_mean,

durnpso = durnpso_mean,

prevsys,

psa),

study = study,

all_contrasts = TRUE) %>%

as_tibble() %>%

mutate(trtb = factor(gsub("^d\\[(.+: )?(.+) vs\\. (.+)\\]$", "\\2", parameter),

levels = levels(pso_net$treatments)),

trta = factor(gsub("^d\\[(.+: )?(.+) vs\\. (.+)\\]$", "\\3", parameter),

levels = levels(pso_net$treatments))) %>%

# Take comparisons against placebo, selected focal comparisons

filter(trta == "PBO" |

(trta %in% c("IXE Q2W", "SEC 300", "UST") &

trtb %in% c("IXE Q2W", "SEC 300", "UST"))) %>%

# Format estimates and CrIs

transmute(trtb, trta,

`Study population` = .study,

Contrast = paste0(trtb, " vs.\\ ", trta),

interval = if_else(is.na(mean), "",

paste0("(", tabfmt(`2.5%`), ", ",

tabfmt(`97.5%`), ")")

),

est = if_else(is.na(mean), "-", tabfmt(mean))

) %>%

# Transpose into table

gather(stat, value, est, interval) %>%

pivot_wider(names_from = `Study population`, values_from = value) %>%

arrange(trta, trtb, stat) %>%

group_by(Contrast) %>% mutate(grpid = 1:n()) %>% ungroup() %>%

mutate(Contrast = if_else(grpid == 1, Contrast, "")) %>%

select(-stat, -trta, -trtb, -grpid) %>%

# Output to Latex

xtable() %>%

print(file = "./tables/pso_multi_contrasts_targetpops.tex",

sanitize.colnames.function = function(x) {x},

sanitize.text.function = function(x) {x},

add.to.row = list(pos = as.list(seq(2, nrow(.), 2)), command = rep("[1.5ex]", nrow(.)/2)),

include.rownames = FALSE,

booktabs = TRUE,

only.contents = TRUE,

comment = FALSE)

## Figure comparing population-average relative effects -------------------

pso_releff_FE %>%

as_tibble() %>%

mutate(.trt = factor(gsub("^d\\[(.+: )?(.+)\\]$", "\\2", parameter),

levels = levels(pso_net$treatments))) %>%

ggplot(aes(x = mean, xmin = `2.5%`, xmax = `97.5%`,

y = forcats::fct_rev(.trt))) +

geom_vline(xintercept = 0, size = 0.25) +

geom_errorbar(position = position_dodge(width = 0.75), width = 0, size = 0.3) +

geom_point(position = position_dodge(width = 0.75), fill = "white") +

xlab("SMD (95% CrI)") + ylab("Treatment") +

facet_wrap(~.study) +

theme_multinma(base_family = "Source Sans Pro")

ggsave("./figures/pso_multi_contrasts.pdf", width = 6, height = 4, scale = 1.2)

## Figure comparing population-average relative effects (target pops) -----

pso_releff_FE_new %>%

as_tibble() %>%

mutate(.trt = factor(gsub("^d\\[(.+: )?(.+)\\]$", "\\2", parameter),

levels = levels(pso_net$treatments))) %>%

ggplot(aes(x = mean, xmin = `2.5%`, xmax = `97.5%`,

y = forcats::fct_rev(.trt))) +

geom_vline(xintercept = 0, size = 0.25) +

geom_errorbar(position = position_dodge(width = 0.75), width = 0, size = 0.3) +

geom_point(position = position_dodge(width = 0.75), fill = "white") +

xlab("SMD (95% CrI)") + ylab("Treatment") +

facet_wrap(~.study) +

theme_multinma(base_family = "Source Sans Pro")

ggsave("./figures/pso_multi_contrasts_targetpops.pdf", width = 6, height = 2, scale = 1.2)

## Figure comparing population-average outcome probabilities -------------

# Get model predictions

pso_pred_mod <-

pso_pred_FE %>%

as_tibble() %>%

mutate(method = "ML-NMR",

outcomef = recode_factor(gsub("^pred\\[.+: (.+), PASI(75|90|100)\\]$", "\\2", parameter),

"75" = "PASI 75",

"90" = "PASI 90",

"100" = "PASI 100"),

.trt = factor(gsub("^pred\\[.+: (.+?)(, PASI(75|90|100))?\\]$", "\\1", parameter),

levels = levels(pso_net$treatments)),

estimate = mean, conf.low = `2.5%`, conf.high = `97.5%`)

# Get observed proportions

get_binom_ci <- function(r, n) {

bt <- binom.test(r, n)

tibble(estimate = bt$estimate,

conf.low = bt$conf.int[1],

conf.high = bt$conf.int[2])

}

pso_pred_obs <-

bind_rows(

pso_ipd %>%

pivot_longer(matches("pasi(75|90|100)_w12_nri_01"), names_to = "outcome", values_to = "value",

names_pattern = "(pasi(?:75|90|100))") %>%

group_by(study, trtc, outcome) %>%

summarise(get_binom_ci(sum(value), n())),

pso_agd %>%

pivot_longer(matches("pasi(75|90|100)_r"), names_to = "outcome", values_to = "value",

names_pattern = "(pasi(?:75|90|100))") %>%

group_by(study, trtc, outcome) %>%

summarise(get_binom_ci(value, pasi75_n))

) %>%

mutate(method = "Observed",

outcomef = recode_factor(outcome,

pasi75 = "PASI 75",

pasi90 = "PASI 90",

pasi100 = "PASI 100"),

.trt = factor(trtc, levels = levels(pso_net$treatments)),

.study = factor(study, levels = levels(pso_net$studies)))

pso_pred_all <- bind_rows(pso_pred_mod, pso_pred_obs) %>%

mutate(methodf = factor(method, levels = c("ML-NMR", "Observed")))

# Plot

ggplot(pso_pred_all,

aes(x = estimate*100, xmin = conf.low*100, xmax = conf.high*100,

y = fct_rev(.trt), shape = methodf, colour = methodf)) +

geom_errorbar(width = 0, position = position_dodge(width = 0.75), size = 0.3) +

geom_point(position = position_dodge(width = 0.75), fill = "white") +

xlab("Percent achieving PASI outcome") + ylab("Treatment") +

coord_cartesian(xlim = c(0, 100)) +

facet_grid(.study~outcomef) +

scale_shape_manual("Method", values = c(`ML-NMR` = 16, Observed = 21)) +

scale_colour_manual("Method", values = c(`ML-NMR` = "#00468B", Observed = "#8B0000")) +

theme_multinma(base_family = "Source Sans Pro") +

theme(legend.position = "top",

legend.margin = margin(0, 0, 0, 0),

legend.box.margin = margin(0, 0, -0.5, 0, "lines"))

ggsave("./figures/pso_multi_percent_pasi.pdf", width = 5, height = 8, scale = 1.2)

## Figure comparing population-average outcome probabilities (target pops) -----------

# Get model predictions

pso_pred_mod_new <-

pso_pred_FE_new %>%

as_tibble() %>%

mutate(method = "ML-NMR",

outcomef = recode_factor(gsub("^pred\\[.+: (.+), PASI(75|90|100)\\]$", "\\2", parameter),

"75" = "PASI 75",

"90" = "PASI 90",

"100" = "PASI 100"),

.trt = factor(gsub("^pred\\[.+: (.+?)(, PASI(75|90|100))?\\]$", "\\1", parameter),

levels = levels(pso_net$treatments)),

estimate = mean, conf.low = `2.5%`, conf.high = `97.5%`)

# Get observed proportions

pso_pred_obs_new <-

tribble(~study, ~pasi75_r, ~pasi90_r, ~pasi100_r, ~sample_size,

"Chiricozzi 2019", 243, 127, 71, 330,

"PROSPECT", 1156, 801, 337, 1509) %>%

pivot_longer(matches("pasi(75|90|100)_r"), names_to = "outcome", values_to = "value",

names_pattern = "(pasi(?:75|90|100))") %>%

group_by(study, outcome) %>%

summarise(get_binom_ci(value, sample_size)) %>%

mutate(method = "Observed",

outcomef = recode_factor(outcome,

pasi75 = "PASI 75",

pasi90 = "PASI 90",

pasi100 = "PASI 100"),

.trt = factor("SEC 300", levels = levels(pso_net$treatments)),

.study = factor(study))

pso_pred_all_new <- bind_rows(pso_pred_mod_new, pso_pred_obs_new) %>%

mutate(methodf = factor(method, levels = c("ML-NMR", "Observed")))

# Plot

ggplot(pso_pred_all_new,

aes(x = estimate*100, xmin = conf.low*100, xmax = conf.high*100,

y = fct_rev(.trt), shape = methodf, colour = methodf)) +

geom_errorbar(width = 0, position = position_dodge(width = 0.75), size = 0.3) +

geom_point(position = position_dodge(width = 0.75), fill = "white") +

xlab("Percent achieving PASI outcome") + ylab("Treatment") +

coord_cartesian(xlim = c(0, 100)) +

facet_grid(.study~outcomef) +

scale_shape_manual("Method", values = c(`ML-NMR` = 16, Observed = 21)) +

scale_colour_manual("Method", values = c(`ML-NMR` = "#00468B", Observed = "#8B0000")) +

theme_multinma(base_family = "Source Sans Pro") +

theme(legend.position = "top",

legend.margin = margin(0, 0, 0, 0),

legend.box.margin = margin(0, 0, -0.5, 0, "lines"))

ggsave("./figures/pso_multi_percent_pasi_targetpops.pdf", width = 5, height = 2.5, scale = 1.2)

## Tables of predicted outcome probabilities ------------------------------

pso_pred_tabdat <-

pso_pred_mod %>%

filter(method == "ML-NMR (all PASI cutoffs)") %>%

# Format estimates

transmute(`Study population` = .study,

outcomef,

trt_long = recode_factor(.trt,

PBO = "Placebo",

ETN = "Etanercept",

"IXE Q2W" = "Ixekizumab Q2W",

"IXE Q4W" = "Ixekizumab Q4W",

"SEC 150" = "Secukinumab 150 mg",

"SEC 300" = "Secukinumab 300 mg",

UST = "Ustekinumab"),

`95% CrI` =

if_else(is.na(estimate), "",

paste0("(", tabfmt(conf.low*100), ", ",

tabfmt(conf.high*100), ")")),

Estimate =

if_else(is.na(estimate), "-",

tabfmt(estimate*100))

) %>%

pivot_longer(cols = c(Estimate, `95% CrI`), names_to = "stat", values_to = "value") %>%

pivot_wider(names_from = trt_long, values_from = value) %>%

select(outcomef, `Study population`, stat,

Placebo, Etanercept, `Ixekizumab Q2W`, `Ixekizumab Q4W`,

`Secukinumab 150 mg`, `Secukinumab 300 mg`, Ustekinumab) %>%

arrange(outcomef, `Study population`, desc(stat)) %>%

group_by(outcomef, `Study population`) %>%

mutate(grpid = 1:n()) %>%

ungroup() %>%

mutate(`Study population` = if_else(grpid == 1, as.character(`Study population`), NA_character_)) %>%

select(-stat, -grpid)

for (oc in c("PASI 75", "PASI 90", "PASI 100")) {

pso_pred_tabdat %>%

filter(outcomef == oc) %>%

select(-outcomef) %>%

xtable() %>%

print(file = paste0("./tables/pso_multi_percent_",

tolower(str_replace(oc, " ", "_")),

".tex"),

sanitize.colnames.function = function(x) {x},

sanitize.text.function = function(x) {x},

add.to.row = list(pos = as.list(seq(2, 16, 2)), command = rep("[1.5ex]", 8)),

include.rownames = FALSE,

booktabs = TRUE,

only.contents = TRUE,

comment = FALSE)

}

## Tables of predicted outcome probabilities (target pops) ----------------

pso_pred_mod_new %>%

filter(method == "ML-NMR (all PASI cutoffs)") %>%

# Format estimates

transmute(study = .study,

outcomef,

trt_long = recode_factor(.trt,

PBO = "Placebo",

ETN = "Etanercept",

"IXE Q2W" = "Ixekizumab Q2W",

"IXE Q4W" = "Ixekizumab Q4W",

"SEC 150" = "Secukinumab 150 mg",

"SEC 300" = "Secukinumab 300 mg",

UST = "Ustekinumab"),

`95% CrI` =

if_else(is.na(estimate), "",

paste0("(", tabfmt(conf.low*100), ", ",

tabfmt(conf.high*100), ")")),

Estimate =

if_else(is.na(estimate), "-",

tabfmt(estimate*100))

) %>%

pivot_longer(cols = c(Estimate, `95% CrI`), names_to = "stat", values_to = "value") %>%

pivot_wider(names_from = trt_long, values_from = value) %>%

select(outcomef, study, stat,

Placebo, Etanercept, `Ixekizumab Q2W`, `Ixekizumab Q4W`,

`Secukinumab 150 mg`, `Secukinumab 300 mg`, Ustekinumab) %>%

arrange(study, outcomef, desc(stat)) %>%

group_by(study, outcomef) %>%

mutate(grpid = 1:n()) %>%

ungroup() %>%

mutate(Population = if_else(grpid == 1, as.character(outcomef), NA_character_), .before = 1) %>%

group_by(study) %>%

group_modify(~add_row(.x, Population = paste0("\\rowgroup{", pull(.y), "}"), .before = 1)) %>%

ungroup() %>%

rename("\\rowgroup{Population}" = Population) %>%

select(-stat, -grpid, -study, -outcomef) %>%

# Output to latex

xtable() %>%

print(file = paste0("./tables/pso_multi_percent_targetpops.tex"),

sanitize.colnames.function = function(x) {x},

sanitize.text.function = function(x) {x},

add.to.row = list(pos = as.list(c(1, 3, 5, 7, 8, 10, 12, 14)), command = rep("[1.5ex]", 8)),

hline.after = c(-1, 0, 7, nrow(.)),

include.rownames = FALSE,

booktabs = TRUE,

only.contents = TRUE,

comment = FALSE)

# Outputs for independent weight interactions ----------------------------

## Figure comparing relative effects relaxing shared EM -------------------

# Figure comparing population-average relative effects under shared EM model

# (common interactions) and model with independent weight interactions

pso_releff_FE %>%

as_tibble() %>%

mutate(model = "Common") %>%

# Add in independent weight interactions analysis

bind_rows(

relative_effects(pso_fit_FE_SEM_weight) %>%

as_tibble() %>%

mutate(model = "Independent")

) %>%

mutate(.trt = factor(gsub("^d\\[(.+: )?(.+)\\]$", "\\2", parameter),

levels = levels(pso_net$treatments))) %>%

ggplot(aes(x = mean, xmin = `2.5%`, xmax = `97.5%`,

y = forcats::fct_rev(.trt), colour = model, shape = model)) +

geom_vline(xintercept = 0, size = 0.25) +

geom_errorbar(position = position_dodge(width = 0.75), width = 0, size = 0.3) +

geom_point(position = position_dodge(width = 0.75), fill = "white") +

xlab("SMD (95% CrI)") + ylab("Treatment") +

facet_wrap(~.study) +

scale_shape_manual("Interactions", values = c(Common = 16, Independent = 22)) +

scale_colour_manual("Interactions", values = c(Common = "#00468B", Independent = "#008B46")) +

theme_multinma(base_family = "Source Sans Pro") +

theme(legend.position = "top",

legend.margin = margin(0, 0, 0, 0),

legend.box.margin = margin(0, 0, -0.5, 0, "lines"))

ggsave("./figures/pso_multi_contrasts_SEM_weight.pdf", width = 6, height = 4, scale = 1.2)

## Figure comparing relative effects relaxing shared EM (target pops) -----

# Figure comparing population-average relative effects under shared EM model

# (common interactions) and model with independent weight interactions

pso_releff_FE_new %>%

as_tibble() %>%

mutate(model = "Common") %>%

# Add in independent weight interactions analysis

bind_rows(

relative_effects(pso_fit_FE_SEM_weight,

newdata = transmute(new_agd_means_rs,

study,

bsa = bsa_mean,

weight = weight_mean,

durnpso = durnpso_mean,

prevsys,

psa),

study = study) %>%

as_tibble() %>%

mutate(model = "Independent")

) %>%

mutate(.trt = factor(gsub("^d\\[(.+: )?(.+)\\]$", "\\2", parameter),

levels = levels(pso_net$treatments))) %>%

ggplot(aes(x = mean, xmin = `2.5%`, xmax = `97.5%`,

y = forcats::fct_rev(.trt), colour = model, shape = model)) +

geom_vline(xintercept = 0, size = 0.25) +

geom_errorbar(position = position_dodge(width = 0.75), width = 0, size = 0.3) +

geom_point(position = position_dodge(width = 0.75), fill = "white") +

xlab("SMD (95% CrI)") + ylab("Treatment") +

facet_wrap(~.study) +

scale_shape_manual("Interactions", values = c(Common = 16, Independent = 22)) +

scale_colour_manual("Interactions", values = c(Common = "#00468B", Independent = "#008B46")) +

theme_multinma(base_family = "Source Sans Pro") +

theme(legend.position = "top",

legend.margin = margin(0, 0, 0, 0),

legend.box.margin = margin(0, 0, -0.5, 0, "lines"))

ggsave("./figures/pso_multi_contrasts_SEM_weight_targetpops.pdf", width = 6, height = 2, scale = 1.2)

## Figure comparing predicted probabilities relaxing shared EM -------------------

# Figure comparing population-average outcome probabilities under shared EM

# model (common interactions) and model with independent weight interactions

# Get model predictions

pso_pred_FE_SEM_weight_save <- "./rds/multi_pso_pred_FE_SEM_weight.rds"

if (interactive() && file.exists(pso_pred_FE_SEM_weight_save) && toupper(readline("Read in saved results? [y/n]: ")) == "Y") {

pso_pred_FE_SEM_weight <- readRDS(pso_pred_FE_SEM_weight_save)

} else {

pso_pred_FE_SEM_weight <- predict(pso_fit_FE_SEM_weight, type = "response")

saveRDS(pso_pred_FE_SEM_weight, pso_pred_FE_SEM_weight_save)

}

pso_pred_mod_SEM_weight <-

bind_rows(

pso_pred_FE %>%

as_tibble() %>%

mutate(method = "Common interactions") ,

pso_pred_FE_SEM_weight %>%

as_tibble() %>%

mutate(method = "Independent interactions")

) %>%

mutate(.trt = factor(gsub("^pred\\[.+: (.+?)(, PASI(75|90|100))?\\]$", "\\1", parameter),

levels = levels(pso_net$treatments)),

outcomef = recode_factor(gsub("^pred\\[.+: (.+), PASI(75|90|100)\\]$", "\\2", parameter),

"75" = "PASI 75",

"90" = "PASI 90",

"100" = "PASI 100"),

estimate = mean, conf.low = `2.5%`, conf.high = `97.5%`)

pso_pred_all_SEM_weight <- bind_rows(pso_pred_mod_SEM_weight, pso_pred_obs) %>%

mutate(methodf = factor(method, levels = c("Common interactions", "Independent interactions", "Observed")))

# Plot

ggplot(pso_pred_all_SEM_weight,

aes(x = estimate*100, xmin = conf.low*100, xmax = conf.high*100,

y = fct_rev(.trt), shape = methodf, colour = methodf)) +

geom_errorbar(position = position_dodge(width = 0.75), width = 0, size = 0.3) +

geom_point(position = position_dodge(width = 0.75), fill = "white") +

xlab("Percent achieving PASI outcome") + ylab("Treatment") +

coord_cartesian(xlim = c(0, 100)) +

facet_grid(.study~outcomef) +

scale_shape_manual("Method", values = c(`Common interactions` = 16,

`Independent interactions` = 22,

Observed = 21)) +

scale_colour_manual("Method", values = c(`Common interactions` = "#00468B",

`Independent interactions` = "#008B46",

Observed = "#8B0000")) +

theme_multinma(base_family = "Source Sans Pro") +

theme(legend.position = "top",

legend.margin = margin(0, 0, 0, 0),

legend.box.margin = margin(0, 0, -0.5, 0, "lines"))

ggsave("./figures/pso_multi_percent_pasi_SEM_weight.pdf", width = 5, height = 8, scale = 1.2)

## Figure comparing predicted probabilities relaxing shared EM (target pops) -----

pso_pred_FE_SEM_weight_new <- predict(pso_fit_FE_SEM_weight,

type = "response",

newdata = new_agd_int,

study = study,

baseline = list(PROSPECT = distr(qbeta, 1156, 1509-1156),

"Chiricozzi 2019" = distr(qbeta, 243, 330-243)),

baseline_type = "response",

baseline_level = "aggregate",

trt_ref = "SEC 300")

pso_pred_mod_SEM_weight_new <-

bind_rows(

pso_pred_FE_new %>%

as_tibble() %>%

mutate(method = "Common interactions"),

pso_pred_FE_SEM_weight_new %>%

as_tibble() %>%

mutate(method = "Independent interactions")

) %>%

mutate(.trt = factor(gsub("^pred\\[.+: (.+?)(, PASI(75|90|100))?\\]$", "\\1", parameter),

levels = levels(pso_net$treatments)),

outcomef = recode_factor(gsub("^pred\\[.+: (.+), PASI(75|90|100)\\]$", "\\2", parameter),

"75" = "PASI 75",

"90" = "PASI 90",

"100" = "PASI 100"),

estimate = mean, conf.low = `2.5%`, conf.high = `97.5%`)

pso_pred_all_SEM_weight_new <- bind_rows(pso_pred_mod_SEM_weight_new, pso_pred_obs_new) %>%

mutate(methodf = factor(method, levels = c("Common interactions", "Independent interactions", "Observed")))

# Plot

ggplot(pso_pred_all_SEM_weight_new,

aes(x = estimate*100, xmin = conf.low*100, xmax = conf.high*100,

y = fct_rev(.trt), shape = methodf, colour = methodf)) +

geom_errorbar(position = position_dodge(width = 0.75), width = 0, size = 0.3) +

geom_point(position = position_dodge(width = 0.75), fill = "white") +

xlab("Percent achieving PASI outcome") + ylab("Treatment") +

coord_cartesian(xlim = c(0, 100)) +

facet_grid(.study~outcomef) +

scale_shape_manual("Method", values = c(`Common interactions` = 16,

`Independent interactions` = 22,

Observed = 21))

scale_colour_manual("Method", values = c(`Common interactions` = "#00468B",

`Independent interactions` = "#008B46",

Observed = "#8B0000")) +

theme_multinma(base_family = "Source Sans Pro") +

theme(legend.position = "top",

legend.margin = margin(0, 0, 0, 0),

legend.box.margin = margin(0, 0, -0.5, 0, "lines"))

ggsave("./figures/pso_multi_percent_pasi_SEM_weight_targetpops.pdf", width = 5, height = 2.5, scale = 1.2)

# Sensitivity analyses ----------------------------------------------------

# Sensitivity analyses for models omitting covariates

mods <- list(pso_fit_FE_nodurnpso = ~(prevsys + bsa + weight + psa)*.trt,

pso_fit_FE_noprevsys = ~(durnpso + bsa + weight + psa)*.trt,

pso_fit_FE_nobsa = ~(durnpso + prevsys + weight + psa)*.trt,

pso_fit_FE_noweight = ~(durnpso + prevsys + bsa + psa)*.trt,

pso_fit_FE_nopsa = ~(durnpso + prevsys + bsa + weight)*.trt)

sfits <- mods

reuse_saved <- TRUE

for (m in 1:length(mods)) {

.save <- paste0("./rds/multi_pso_fit_FE", names(mods)[m], ".rds")

if (file.exists(.save) && (reuse_saved || (interactive() && toupper(readline("Read in saved model fit? [y/n]: ")) == "Y"))) {

cat("Reading saved model", names(mods)[m], "\n")

sfit <- readRDS(.save)

rstan::check_hmc_diagnostics(as.stanfit(sfit))

} else {

cat("Fitting model", names(mods)[m], "\n")

sfit <- nma(pso_net,

trt_effects = "fixed",

link = "probit",

regression = mods[[m]],

class_interactions = "common",

prior_intercept = normal(scale = 10),

prior_trt = normal(scale = 10),

prior_reg = normal(scale = 10),

prior_aux = flat(),

QR = TRUE,

init_r = 0.5,

save_warmup = FALSE)

saveRDS(sfit, file = .save)

}

sfits[[m]] <- sfit

}

lapply(sfits, dic)

# Best fitting model is one omitting BSA

pso_fit_FE_nobsa <- sfits[["pso_fit_FE_nobsa"]]

# Relative effects

(pso_releff_FE_nobsa <- relative_effects(pso_fit_FE_nobsa))

plot(pso_releff_FE_nobsa, ref_line = 0) +

facet_wrap("Study")

# Predicted probabilities

pso_pred_FE_nobsa_save <- "./rds/multi_pso_pred_FE_nobsa.rds"

if (interactive() && file.exists(pso_pred_FE_nobsa_save) && toupper(readline("Read in saved results? [y/n]: ")) == "Y") {

pso_pred_FE_nobsa <- readRDS(pso_pred_FE_nobsa_save)

} else {

pso_pred_FE_nobsa <- predict(pso_fit_FE_nobsa, type = "response")

saveRDS(pso_pred_FE_nobsa, pso_pred_FE_nobsa_save)

}

pso_pred_FE_nobsa

plot(pso_pred_FE_nobsa)

# Relative effects in new target population

(pso_releff_FE_nobsa_new <- relative_effects(pso_fit_FE_nobsa,

newdata = transmute(new_agd_means_rs,

study,

bsa = bsa_mean,

weight = weight_mean,

durnpso = durnpso_mean,

prevsys,

psa),

study = study))

plot(pso_releff_FE_nobsa_new, ref_line = 0) + facet_wrap("Study")

# Predicted probabilities in new target population

(pso_pred_FE_nobsa_new <- predict(pso_fit_FE_nobsa,

type = "response",

newdata = new_agd_int,

study = study,

baseline = list(PROSPECT = distr(qbeta, 1156, 1509-1156),

"Chiricozzi 2019" = distr(qbeta, 243, 330-243)),

baseline_type = "response",

baseline_level = "aggregate",

trt_ref = "SEC 300"))

plot(pso_pred_FE_nobsa_new, ref_line = c(0, 1)) +

facet_wrap("Study") +

aes(colour = Category) +

scale_colour_brewer(palette = "Blues")
