# Supplementary material for: Validating the Assumptions of Population Adjustment: Application of Multilevel Network Meta-regression to a Network of Treatments for Plaque Psoriasis
Source: Med Decis Making. 2022 Aug 23;43(1):53–67. doi: 10.1177/0272989X221117162 (PMC9742635; doi:10.1177/0272989X221117162)
Supplement: sj-pdf-1-mdm-10.1177_0272989X221117162 – Supplemental material for Validating the Assumptions of Population Adjustment: Application of Multilevel Network Meta-regression to a Network of Treatments for Plaque Psoriasis [file sj-pdf-1-mdm-10.1177_0272989X221117162.pdf]

A Appendix

A.1 Statistical methods

A.1.1 ML-NMR for ordered categorical outcomes

With ordered categorical outcomes in M categories, the IPD outcomes are given by $y_{ijk} \in \{1, \ldots, M\}$. For the plaque psoriasis network, there are $M = 4$ categories, and we let $y_{ijk} \in \{1, \ldots, 4\}$ correspond to less than 75% reduction in PASI score (i.e. failure to achieve PASI 75), $\geq 75\%$ and $< 90\%$ reduction (achieving PASI 75 but not PASI 90), $\geq 90\%$ and $< 100\%$ reduction (achieving PASI 90 but not PASI 100), and 100% reduction (achieving PASI 100), respectively, for an individual i in study j receiving treatment k. The individual-level model for ordered categorical outcomes is given by

$$y_{ijk} \sim \text{Categorical}(p_{ijk;1}, \ldots, p_{ijk;M}) \tag{A.1a}$$

$$p_{ijk;m} = \Phi(\eta_{jk}(\boldsymbol{x}_{ijk}) - c_{m-1}) - \Phi(\eta_{jk}(\boldsymbol{x}_{ijk}) - c_m) \quad \text{for } m = 1, \ldots, M \tag{A.1b}$$

$$\eta_{jk}(\boldsymbol{x}_{ijk}) = \mu_j + \boldsymbol{x}_{ijk}^{\mathsf{T}}(\boldsymbol{\beta}_1 + \boldsymbol{\beta}_{2,k}) + \gamma_k \tag{A.1c}$$

where the event probability in each category is $p_{ijk;m}$ and $\sum_{m=1}^{4} p_{ijk,m} = 1$ for each i, j, k. The category event probabilities are transformed onto the linear predictor scale using the probit link function $\Phi^{-1}(\cdot)$ (the Normal inverse cumulative distribution function). We use the probit link function here for comparability with previous analyses, but another suitable choice would be the logit link function. The linear predictor for an individual on treatment k in trial j with covariate vector $\boldsymbol{x}_{ijk}$ is $\eta_{jk}(\boldsymbol{x}_{ijk})$. The parameters μ_j are study-specific baselines, $\boldsymbol{\beta}_1$ are coefficients for prognostic variables, and $\boldsymbol{\beta}_{2,k}$ are coefficients for effect modifiers specific to each treatment k. The effect of treatment k (at the individual level), γ_k, is defined with respect to the network reference treatment 1, and we set $\gamma_1 = 0$ and $\boldsymbol{\beta}_{2,1} = \mathbf{0}$. Some coefficients in $\boldsymbol{\beta}_1$ or $\boldsymbol{\beta}_{2,k}$ may be set to zero, if it is known that a particular covariate is not prognostic or effect modifying respectively. The consistency equations on the individual-level treatment effects, where the relative effect of treatment b vs. a is $\gamma_{ab} = \gamma_b - \gamma_a$, are implicit in this model formulation, which is equivalent to the widely-used "baseline shift" formulation of NMA models [4]. A similar set of consistency equations are also implicit on the effect modifier interactions $\boldsymbol{\beta}_{2,ab} = \boldsymbol{\beta}_{2,b} - \boldsymbol{\beta}_{2,a}$. The latent

cutpoints between categories c_m are subject to the ordering constraints

$$c_0 < c_1 < \cdots < c_M, \tag{A.2}$$

and, we set $c_0 = -\infty$, $c_1 = 0$, and $c_M = +\infty$. For the plaque psoriasis analysis, the latent cutpoint c_1 corresponds to achieving PASI 75, c_2 corresponds to achieving PASI 90, and c_3 corresponds to achieving PASI 100. A very similar model has previously been proposed for standard AgD NMA of ordered count data [4, 27]; indeed, model (A.1) reduces to this when there are no covariates.

Priors for the latent cutpoints are most straightforward to specify on the differences between adjacent cutpoints, for example $c_m - c_{m-1} \sim \mathrm{U}(0, u_c)$ for $m = 2, \ldots, M-1$ with an appropriate upper bound u_c (as used by Dias et al. [4] with $u_c = 5$), so that the ordering constraints (A.2) are satisfied. When fitting the model in Stan, the ordering constraints (A.2) are guaranteed by declaring the c_m to be an `ordered` vector, so prior distributions can be placed directly on the cutpoints if desired. In this analysis, we place improper uniform prior distributions $\mathrm{U}(-\infty, +\infty)$ on c_2 and c_3, which are automatically truncated to satisfy the ordering constraints (A.2). We also place vague $\mathrm{N}(0, 10^2)$ prior distributions on each of the parameters μ_j, $\boldsymbol{\beta}_1$, $\boldsymbol{\beta}_{2,j}$, and γ_k.

Aggregate outcomes are vectors of summary outcome counts in each category $\boldsymbol{y}_{\bullet jk} = (y_{\bullet jk;1}, \ldots, y_{\bullet jk;M})^{\mathsf{T}}$. We work with these category counts in "exclusive" format, where individuals are only counted in the highest category they achieve (as opposed to "inclusive" counts where individuals are counted in every category up to and including the highest category achieved; it is a straightforward matter of addition or subtraction to convert between formats). These summary data are given a Multinomial likelihood, with the average event probabilities in each category obtained by integrating the individual-level model (A.1b) over the covariate joint distribution $f_{jk}(\cdot)$ in each arm of each AgD study:

$$\boldsymbol{y}_{\bullet jk} \sim \mathrm{Multi}(\bar{p}_{jk;1}, \ldots, \bar{p}_{jk;M}; N_{jk}) \tag{A.3a}$$

$$\bar{p}_{jk;m} = \bar{p}_{jk;m} = \bar{q}_{jk;m-1} - \bar{q}_{jk;m} \quad \text{for } m = 1, \ldots, M, \text{ where} \tag{A.3b}$$

$$\bar{q}_{jk;m} = \int_{\mathfrak{X}} \Phi(\eta_{jk}(\boldsymbol{x}) - c_m) f_{jk}(\boldsymbol{x})\, d\boldsymbol{x} \tag{A.3c}$$

where $\bar{p}_{jk;m}$ are the average event probabilities in each category and $N_{jk} = \sum_k y_{\bullet jk;m}$ is is the sample size in each arm. We compute the integrals for $\bar{q}_{jk;m}$ in (A.3c) using Quasi-Monte Carlo integration [13] with $\tilde{N} = 1000$ integration points $\tilde{\boldsymbol{x}}_{jk}$ drawn from joint distribution $f_{jk}(\cdot)$ of the

covariates on each treatment k in study j, so that

$$\bar{q}_{jk;m} \simeq \tilde{N}^{-1} \sum \Phi(\eta_{jk}(\tilde{\boldsymbol{x}}_{jk}) - c_m).$$

Accounting for studies reporting a subset of categories

It is not uncommon for some studies to report only a subset of categories. These studies can be incorporated at either the individual or aggregate level by modifying equations Equation (A.1) or Equation (A.3), respectively, to involve the relevant latent cutpoints [4]. That is, given a reported set of categories $s_1 < \cdots < s_{M_j}$ in study j, the individual-level event probabilities in (A.1b) for the reported categories become

$$p_{ijk;s_m} = \Phi(\eta_{jk}(\boldsymbol{x}_{ijk}) - c_{s_{m-1}}) - \Phi(\eta_{jk}(\boldsymbol{x}_{ijk}) - c_{s_m}) \quad \text{for } m = 1, \ldots, M_j.$$

Similarly at the aggregate level, the average event probabilities (A.3b) in each of the reported categories become

$$\bar{p}_{jk;s_m} = \bar{p}_{jk;m} = \bar{q}_{jk;s_{m-1}} - \bar{q}_{jk;s_m} \quad \text{for } m = 1, \ldots, M_j.$$

A.1.2 Producing population-average estimates for populations of interest

Population-average estimates of quantities of interest to decision-making, such as average treatment effects and average event probabilities, can be produced by averaging estimates of individual-level quantities over the covariate joint distribution in the target population [13]. For decision-making based on cost-effectiveness models, the typical inputs are the population-average event probabilities for a cohort-based model (e.g. a decision tree or Markov model) or individual event probabilities for an individual-based model (e.g. a discrete event simulation). The target population need not be one of the studies in the network; indeed, it is more likely represented by a registry or cohort study [11].

To estimate the proportion of individuals achieving each PASI endpoint in a given population, we first note that the probability of an individual in population P with covariate values $\boldsymbol{x}$ achieving PASI endpoint m or greater is

$$q_{k;m(P)}(\boldsymbol{x}) = \Phi\big(\mu_{(P)} + \boldsymbol{x}^{\top}(\boldsymbol{\beta}_1 + \boldsymbol{\beta}_{2,k}) + \gamma_k - c_m\big), \tag{A.4}$$

where $m = 1$ corresponds to PASI 75, $m = 2$ to PASI 90, and $m = 3$ to PASI 100; $\mu_{(P)}$ is the baseline probit probability of achieving PASI 75 in population P. A distribution for $\mu_{(P)}$ is required, which may be estimated from external data on the population P. Appendix A.1.3 describes techniques for deriving a distribution for $\mu_{(P)}$ in some common evidence scenarios for baseline response. Here, we produce estimates for each of the observed study populations and simply use the intercepts μ_j estimated in the model. We then average (A.4) over the covariate joint distribution $f_{(P)}(\cdot)$ in population P. This may be performed in general by

$$\bar{q}_{k;m(P)} = N^{-1} \sum_{i=1}^{N} q_{k;m(P)}(\boldsymbol{x}_{i(P)}), \tag{A.5}$$

where $\boldsymbol{x}_{i(P)}$ are N samples from $f_{(P)}(\cdot)$. For example, $\boldsymbol{x}_{i(P)}$ may be $N_{(P)}$ covariate values of individuals in a large representative sample from the population P, or these may be $\tilde{N}$ integration points if the joint covariate distribution in the population has been constructed from summary information or from a small sample of IPD (following section "Using published marginal covariate information").

For quantities on the linear predictor scale such as population-average treatment effects, averaging over the joint covariate distribution is equivalent to "plugging-in" mean covariate values from the population of interest. The population-average treatment effect between treatments a and b in population P is estimated using

$$d_{ab(P)} = \bar{\boldsymbol{x}}_{(P)}^{\top}(\boldsymbol{\beta}_{2,b} - \boldsymbol{\beta}_{2,a}) + \gamma_b - \gamma_a, \tag{A.6}$$

where $\bar{\boldsymbol{x}}_{(P)}$ is the vector of mean covariate values in population P.

The population-average treatment effects $d_{ab(P)}$ produced by equation (A.6) are population-average *conditional* treatment effects, the average effect between randomly selected treated and untreated individuals with the same covariates in the population. On the other hand, MAIC targets the (population-average) *marginal* treatment effect, the average effect between randomly selected treated and untreated individuals in the population, regardless of their covariates. ML-NMR can also produce estimates of these marginal treatment effects, as

$$\Delta_{ab;m(P)} = \Phi^{-1}\big(\bar{q}_{b;m(P)}\big) - \Phi^{-1}\big(\bar{q}_{a;m(P)}\big), \tag{A.7}$$

for any two treatments b and a in population P for outcome category m, where $\bar{q}_{k;m(P)}$ are

estimated using equation (A.5). Analogous approaches can be used to express marginal effects in terms of risk differences or risk ratios for interpretation. Due to non-collapsibility, $\Delta_{ab;m(P)}$ will differ from $d_{ab(P)}$, and—like the average category event probabilities $\bar{q}_{k;m(P)}$—will also differ by the distributions of baseline event probabilities and prognostic (as well as effect-modifying) variables between populations, and between categories. Moreover, rankings based on the marginal effects $\Delta_{ab;m(P)}$ (or equivalently on the average event probabilities $\bar{q}_{k;m(P)}$) may differ from those based on the population-average conditional effects $d_{ab(P)}$ when there is effect modification, because the estimands correspond to different decision questions. For decision-making based on cost-effectiveness models, the typical inputs are the population-average event probabilities $\bar{q}_{k;m(P)}$ for a cohort-based model (e.g. a decision tree or Markov model) or individual event probabilities $q_{k;m(P)}(\boldsymbol{x})$ for an individual-based model (e.g. a discrete event simulation).

A.1.3 Transforming information on baseline response

As described in Appendix A.1.2, to produce estimates of quantities of interest such as predicted probabilities of response we require a distribution for the baseline response probit probability $\mu_{(P)}$ in the target population P. For ML-NMR models the interpretation of $\mu_{(P)}$ is the probit probability of achieving the lowest response category (PASI 75) on the network reference treatment 1, for an individual in population P at the reference value of the covariates. However, we may not have information on baseline response in this format. Here we describe some simple transformations for obtaining the baseline response information in the required format.

For example, if we have baseline response information as a distribution for $\mu^{(k)}_{(P);m}$ the probit probability of response for an individual in P on treatment k in response category m at the reference level of the covariates, we can recover $\mu_{(P)}$ from the relation $\mu_{(P)} = \mu^{(k)}_{(P);m} - \gamma_k + c_m$. This calculation can be performed within the ML-NMR model code at the time of fitting, or afterwards using posterior samples of γ_k and c_m from the fitted model and a sample (of the same size) from the distribution of $\mu^{(k)}_{(P);m}$.

Moreover, it is perhaps more usual to have information on the baseline response probability at the population level, say the average response probability on treatment k in response category m in the population, $\bar{q}_{k;m(P)}$. We can obtain a sample of the distribution for $\mu_{(P)}$ from a sample of the distribution for $\bar{q}_{k;m(P)}$ by solving equation (A.5) for $\mu_{(P)}$. This can be performed using numerical optimisation, either within the ML-NMR model code at the time of fitting or using posterior samples from the model parameters and a sample of the same length for $\bar{q}_{k;m(P)}$, solving

(A.5) at every sample. By definition any link function (including the probit and logit) is smooth and monotonic, so we have a well-behaved optimisation problem with a unique solution.
