# Supplementary material for: Validating the Assumptions of Population Adjustment: Application of Multilevel Network Meta-regression to a Network of Treatments for Plaque Psoriasis
Source: Med Decis Making. 2022 Aug 23;43(1):53–67. doi: 10.1177/0272989X221117162 (PMC9742635; doi:10.1177/0272989X221117162)
Supplement: sj-pdf-2-mdm-10.1177_0272989X221117162 – Supplemental material for Validating the Assumptions of Population Adjustment: Application of Multilevel Network Meta-regression to a Network of Treatments for Plaque Psoriasis [file sj-pdf-2-mdm-10.1177_0272989X221117162.pdf]

A.2 Details of included studies and target populations

Table A.1: Structure of studies and treatments included in the network; • indicates treatment arms included in studies from which IPD were available, ∘ indicates treatment arms included in studies from which AgD were available.

	CLEAR	ERASURE	FEATURE	FIXTURE	IXORA-S	JUNCTURE	UNCOVER-1	UNCOVER-2	UNCOVER-3
Placebo	·	∘	∘	∘	·	∘	•	•	•
Etanercept	·	·	·	∘	·	·	·	•	•
Ixekizumab Q2W	·	·	·	·	•	·	•	•	•
Ixekizumab Q4W	·	·	·	·	·	·	•	•	•
Secukinumab 150 mg	·	∘	∘	∘	·	∘	·	·	·
Secukinumab 300 mg	∘	∘	∘	∘	·	∘	·	·	·
Ustekinumab	∘	·	·	·	•	·	·	·	·

Table A.2: Baseline covariate summaries for each study. Reported sample size for UNCOVER-2 and 3 after removing two individuals from each study with missing weight, and one individual from IXORA-S with missing body surface area. Statistics are mean (SD) unless otherwise specified.
* Covariate considered a potential effect modifier, to be included in population adjustment.

	CLEAR (N = 676)	ERASURE (N = 738)	FEATURE (N = 177)	FIXTURE (N = 1306)	IXORA-S (N = 259)	JUNCTURE (N = 182)	UNCOVER-1 (N = 1296)	UNCOVER-2 (N = 1219)	UNCOVER-3 (N = 1339)
Age, years	44.9 (13.8)	45.1 (13.1)	45.9 (13.9)	44.5 (12.9)	43.7 (12.8)	44.7 (13.8)	45.7 (12.9)	45.0 (13.0)	45.7 (13.1)
* Body surface area, per cent	32.3 (17.3)	31.9 (18.1)	32.0 (17.3)	34.4 (18.9)	29.1 (17.0)	27.4 (14.7)	27.7 (17.3)	26.0 (16.5)	28.3 (17.1)
* Duration of psoriasis, years	17.8 (12.1)	17.4 (11.8)	19.5 (13.0)	16.5 (12.0)	18.0 (11.8)	20.5 (13.4)	19.6 (11.9)	18.7 (12.5)	18.2 (12.2)
Baseline PASI score	21.6 (8.3)	22.1 (9.4)	20.8 (8.2)	23.7 (10.2)	21.3 (8.5)	20.1 (7.3)	20.1 (8.0)	19.6 (7.2)	20.9 (8.2)
* Previous systemic treatment (%)	67.5	63.0	67.2	64.0	92.3	55.5	71.3	64.2	57.1
* Psoriatic arthritis (%)	18.2	23.2	16.4	14.7	14.7	23.1	26.3	23.6	20.5
Male (%)	71.2	69.0	66.1	71.1	67.2	68.7	68.1	67.0	68.2
* Weight, kg	87.3 (21.0)	88.5 (23.8)	91.6 (24.4)	83.3 (20.8)	87.7 (22.3)	91.6 (25.3)	92.2 (23.8)	91.6 (22.2)	91.2 (23.5)

Table A.3: Covariate summaries for each external target population. Statistics are mean (SD) unless otherwise specified.
* Covariate considered a potential effect modifier, to be included in population adjustment.

	Chiricozzi 2019 (N = 330)	PROSPECT (N = 1509)	PsoBest (N = 2556)
Age, years	51.9 (14.6)	48.1 (13.7)	47.4 (14.1)
Baseline PASI score	16.6 (8.2)	17.7 (12.5)	14.7 (9.7)
* Body surface area, per cent	23.0 (16.8)	18.7 (18.4)	24.0 (20.5)
* Duration of psoriasis, years	16.9 (10.8)	19.6 (13.5)	18.2 (14.1)
Male (%)	68.2	62.4	60.1
* Previous systemic treatment (%)	90.6	91.0	54.0
* Psoriatic arthritis (%)	21.5	20.2	20.7
* Weight, kg	78.3 (15.9)	87.5 (20.3)	85.0 (19.1)

A.3 Additional results figures and tables

Table A.4: Estimated population-average treatment effects (standardised mean differences) and 95% Credible Intervals for each treatment compared to placebo, plus selected focal comparisons of interest between licensed dose regimens, in each study population using the ML-NMR model and for the FE NMA.

	ML-NMR study population									
Contrast	CLEAR	ERASURE	FEATURE	FIXTURE	IXORA-S	JUNCTURE	UNCOVER-1	UNCOVER-2	UNCOVER-3	FE NMA
ETN vs. PBO	1.71<br>(1.55, 1.87)	1.68<br>(1.53, 1.84)	1.69<br>(1.53, 1.87)	1.73<br>(1.56, 1.90)	1.71<br>(1.52, 1.93)	1.68<br>(1.51, 1.85)	1.67<br>(1.50, 1.86)	1.65<br>(1.49, 1.82)	1.65<br>(1.49, 1.81)	1.61<br>(1.47, 1.74)
IXE Q2W vs. PBO	2.95<br>(2.78, 3.11)	2.94<br>(2.78, 3.10)	2.95<br>(2.78, 3.12)	2.93<br>(2.77, 3.11)	2.97<br>(2.77, 3.17)	2.96<br>(2.79, 3.13)	2.98<br>(2.80, 3.15)	2.95<br>(2.78, 3.11)	2.92<br>(2.77, 3.08)	2.86<br>(2.73, 3.00)
IXE Q4W vs. PBO	2.78<br>(2.61, 2.94)	2.77<br>(2.61, 2.93)	2.78<br>(2.61, 2.96)	2.76<br>(2.59, 2.94)	2.80<br>(2.60, 3.00)	2.79<br>(2.63, 2.97)	2.81<br>(2.64, 2.98)	2.78<br>(2.62, 2.94)	2.75<br>(2.60, 2.91)	2.68<br>(2.55, 2.83)
SEC 150 vs. PBO	2.31<br>(2.13, 2.49)	2.31<br>(2.13, 2.48)	2.31<br>(2.13, 2.51)	2.30<br>(2.13, 2.48)	2.33<br>(2.12, 2.56)	2.33<br>(2.14, 2.53)	2.34<br>(2.15, 2.55)	2.31<br>(2.12, 2.51)	2.29<br>(2.11, 2.47)	2.18<br>(2.02, 2.33)
SEC 300 vs. PBO	2.72<br>(2.55, 2.90)	2.72<br>(2.54, 2.89)	2.72<br>(2.54, 2.91)	2.71<br>(2.53, 2.88)	2.74<br>(2.53, 2.96)	2.74<br>(2.55, 2.93)	2.75<br>(2.56, 2.95)	2.72<br>(2.54, 2.91)	2.70<br>(2.52, 2.87)	2.58<br>(2.43, 2.73)
UST vs. PBO	2.28<br>(2.05, 2.50)	2.28<br>(2.05, 2.51)	2.28<br>(2.04, 2.51)	2.28<br>(2.05, 2.51)	2.25<br>(2.00, 2.52)	2.28<br>(2.01, 2.56)	2.28<br>(2.04, 2.53)	2.26<br>(2.01, 2.50)	2.26<br>(2.01, 2.51)	2.14<br>(1.95, 2.34)
SEC 300 vs. IXE Q2W	−0.22<br>(−0.39, −0.07)	−0.22<br>(−0.39, −0.07)	−0.22<br>(−0.39, −0.07)	−0.22<br>(−0.39, −0.07)	−0.22<br>(−0.39, −0.07)	−0.22<br>(−0.39, −0.07)	−0.22<br>(−0.39, −0.07)	−0.22<br>(−0.39, −0.07)	−0.22<br>(−0.39, −0.07)	−0.28<br>(−0.43, −0.14)
UST vs. IXE Q2W	−0.67<br>(−0.86, −0.47)	−0.66<br>(−0.87, −0.45)	−0.67<br>(−0.87, −0.47)	−0.66<br>(−0.86, −0.44)	−0.72<br>(−0.92, −0.52)	−0.68<br>(−0.92, −0.43)	−0.69<br>(−0.89, −0.49)	−0.69<br>(−0.90, −0.47)	−0.67<br>(−0.90, −0.43)	−0.72<br>(−0.90, −0.54)
UST vs. SEC 300	−0.44<br>(−0.60, −0.28)	−0.43<br>(−0.61, −0.26)	−0.45<br>(−0.61, −0.28)	−0.43<br>(−0.60, −0.26)	−0.49<br>(−0.69, −0.30)	−0.45<br>(−0.67, −0.24)	−0.47<br>(−0.64, −0.29)	−0.46<br>(−0.65, −0.28)	−0.44<br>(−0.64, −0.24)	−0.44<br>(−0.58, −0.29)

Table A.5: Estimated proportion of individuals achieving PASI 75 on each treatment in each study population, along with 95% Credible Intervals, using ML-NMR combining information from all PASI endpoints. For interpretability, these are given as inclusive probabilities (i.e. the probability of achieving 75% reduction or greater in PASI score).

	Treatment						
Study population	Placebo	Etanercept	Ixekizumab Q2W	Ixekizumab Q4W	Secukinumab 150 mg	Secukinumab 300 mg	Ustekinumab
CLEAR	8.47<br>(5.95, 11.53)	59.64<br>(53.03, 66.12)	92.34<br>(89.19, 94.81)	89.73<br>(85.76, 92.94)	79.60<br>(74.62, 84.04)	88.89<br>(86.17, 91.14)	78.84<br>(75.08, 82.45)
ERASURE	4.33<br>(3.06, 5.80)	45.33<br>(39.56, 51.02)	86.13<br>(82.12, 89.57)	82.25<br>(77.65, 86.39)	68.73<br>(64.94, 72.36)	80.99<br>(77.76, 84.04)	67.07<br>(59.89, 73.89)
FEATURE	4.29<br>(2.57, 6.60)	45.28<br>(37.14, 53.67)	85.62<br>(79.68, 90.57)	81.67<br>(74.92, 87.76)	68.00<br>(60.76, 74.79)	80.40<br>(74.64, 85.59)	66.74<br>(57.24, 75.41)
FIXTURE	3.74<br>(2.67, 5.06)	44.55<br>(40.21, 48.91)	84.83<br>(80.96, 88.15)	80.70<br>(76.17, 84.69)	66.53<br>(62.14, 70.64)	79.35<br>(75.89, 82.48)	64.64<br>(57.70, 71.31)
IXORA-S	4.95<br>(2.89, 7.64)	49.44<br>(41.85, 56.96)	87.22<br>(82.47, 91.10)	83.48<br>(77.67, 88.53)	70.22<br>(62.25, 77.65)	82.25<br>(76.80, 87.00)	70.62<br>(63.16, 77.16)
JUNCTURE	4.33<br>(2.57, 6.61)	44.66<br>(35.96, 53.02)	85.67<br>(79.63, 90.52)	81.74<br>(74.79, 87.62)	68.17<br>(60.70, 74.91)	80.47<br>(74.35, 85.73)	67.21<br>(57.73, 75.97)
UNCOVER-1	4.72<br>(3.49, 6.10)	46.42<br>(41.95, 50.89)	86.91<br>(84.11, 89.36)	83.16<br>(80.05, 86.03)	69.87<br>(63.96, 75.59)	81.85<br>(77.27, 86.11)	69.50<br>(61.95, 76.65)
UNCOVER-2	4.42<br>(3.22, 5.80)	44.31<br>(40.63, 47.88)	85.86<br>(83.27, 88.24)	81.91<br>(78.93, 84.64)	68.09<br>(62.21, 73.71)	80.53<br>(75.95, 84.80)	67.52<br>(59.62, 74.86)
UNCOVER-3	5.93<br>(4.39, 7.63)	50.08<br>(46.73, 53.43)	88.78<br>(86.48, 90.74)	85.40<br>(82.77, 87.69)	73.02<br>(67.56, 78.07)	84.20<br>(79.88, 87.80)	71.92<br>(64.72, 78.50)

Table A.6: Estimated proportion of individuals achieving PASI 90 on each treatment in each study population, along with 95% Credible Intervals, using ML-NMR combining information from all PASI endpoints. For interpretability, these are given as inclusive probabilities (i.e. the probability of achieving 90% reduction or greater in PASI score).

	Treatment						
Study population	Placebo	Etanercept	Ixekizumab Q2W	Ixekizumab Q4W	Secukinumab 150 mg	Secukinumab 300 mg	Ustekinumab
CLEAR	2.12	34.21	78.14	73.06	56.94	71.46	55.22
	(1.33, 3.18)	(28.25, 40.56)	(72.34, 83.22)	(66.39, 79.06)	(50.47, 63.19)	(67.09, 75.38)	(50.31, 60.26)
ERASURE	0.89	22.28	66.93	60.90	43.58	59.02	41.19
	(0.57, 1.30)	(18.09, 26.76)	(61.04, 72.61)	(54.56, 66.97)	(39.41, 47.83)	(54.65, 63.32)	(33.83, 48.92)
FEATURE	0.89	22.33	66.19	60.14	42.86	58.26	40.96
	(0.46, 1.53)	(16.41, 29.05)	(57.53, 74.37)	(51.10, 69.24)	(35.44, 50.47)	(50.66, 65.80)	(31.41, 50.83)
FIXTURE	0.74	21.46	64.68	58.49	41.05	56.56	38.47
	(0.47, 1.09)	(18.23, 24.87)	(58.93, 70.04)	(52.43, 64.39)	(36.23, 45.98)	(51.72, 61.19)	(31.51, 45.57)
IXORA-S	1.06	25.38	68.48	62.47	45.02	60.59	45.06
	(0.52, 1.86)	(19.53, 31.81)	(60.76, 75.38)	(53.87, 70.27)	(36.32, 53.96)	(52.72, 67.93)	(36.97, 53.05)
JUNCTURE	0.90	21.94	66.37	60.35	43.13	58.47	41.54
	(0.45, 1.56)	(15.77, 28.57)	(57.62, 74.46)	(51.06, 69.28)	(35.30, 50.86)	(50.43, 66.13)	(31.83, 51.58)
UNCOVER-1	1.01	23.13	68.16	62.19	44.89	60.28	43.90
	(0.68, 1.41)	(19.79, 26.71)	(64.37, 71.78)	(58.39, 66.16)	(38.55, 51.55)	(54.13, 66.41)	(35.98, 52.26)
UNCOVER-2	0.92	21.33	66.33	60.23	42.80	58.29	41.61
	(0.60, 1.32)	(18.63, 24.12)	(62.83, 69.87)	(56.40, 63.94)	(36.63, 49.12)	(52.15, 64.49)	(33.60, 50.02)
UNCOVER-3	1.34	25.86	71.41	65.68	48.57	63.82	46.67
	(0.89, 1.88)	(23.12, 28.66)	(67.93, 74.50)	(61.91, 69.08)	(42.24, 54.84)	(57.61, 69.50)	(38.76, 54.91)

Table A.7: Estimated proportion of individuals achieving PASI 100 on each treatment in each study population, along with 95% Credible Intervals, using ML-NMR combining information from all PASI endpoints.

	Treatment						
Study population	Placebo	Etanercept	Ixekizumab Q2W	Ixekizumab Q4W	Secukinumab 150 mg	Secukinumab 300 mg	Ustekinumab
CLEAR	0.23<br>(0.12, 0.40)	11.40<br>(8.41, 14.97)	49.15<br>(41.88, 56.31)	42.73<br>(35.44, 50.13)	26.63<br>(21.43, 32.19)	40.78<br>(36.21, 45.46)	24.53<br>(20.72, 28.75)
ERASURE	0.08<br>(0.04, 0.13)	5.99<br>(4.30, 7.90)	36.09<br>(30.24, 42.08)	30.25<br>(24.70, 36.00)	16.93<br>(14.00, 20.09)	28.52<br>(24.52, 32.58)	14.98<br>(10.79, 19.85)
FEATURE	0.08<br>(0.03, 0.15)	6.05<br>(3.77, 9.00)	35.43<br>(27.29, 44.31)	29.65<br>(22.18, 38.24)	16.52<br>(11.94, 21.70)	27.93<br>(21.69, 34.75)	14.93<br>(9.66, 21.34)
FIXTURE	0.06<br>(0.03, 0.10)	5.56<br>(4.28, 7.03)	33.65<br>(28.16, 39.23)	27.96<br>(22.82, 33.41)	15.26<br>(12.21, 18.76)	26.30<br>(22.09, 30.84)	13.30<br>(9.66, 17.54)
IXORA-S	0.09<br>(0.04, 0.19)	7.20<br>(4.76, 10.18)	37.35<br>(29.72, 45.30)	31.37<br>(23.92, 39.42)	17.67<br>(12.30, 24.12)	29.61<br>(23.08, 36.81)	17.31<br>(12.42, 22.81)
JUNCTURE	0.08<br>(0.03, 0.15)	5.93<br>(3.60, 8.82)	35.71<br>(27.15, 44.62)	29.93<br>(22.14, 38.40)	16.76<br>(11.79, 22.24)	28.22<br>(21.58, 35.30)	15.32<br>(9.94, 21.93)
UNCOVER-1	0.09<br>(0.05, 0.14)	6.32<br>(4.96, 7.88)	37.29<br>(33.96, 40.95)	31.35<br>(28.31, 34.81)	17.80<br>(13.78, 22.43)	29.66<br>(24.47, 35.33)	16.67<br>(11.90, 22.30)
UNCOVER-2	0.08<br>(0.04, 0.13)	5.52<br>(4.48, 6.69)	35.28<br>(31.91, 38.94)	29.45<br>(26.12, 32.81)	16.39<br>(12.51, 20.74)	27.81<br>(22.64, 33.32)	15.18<br>(10.62, 20.56)
UNCOVER-3	0.13<br>(0.07, 0.20)	7.40<br>(6.13, 8.77)	40.85<br>(37.17, 44.53)	34.70<br>(31.15, 38.47)	20.29<br>(15.81, 25.34)	32.93<br>(27.22, 38.97)	18.44<br>(13.41, 24.32)

Table A.8: Estimated population-average treatment effects (standardised mean differences) and 95% Credible Intervals for each treatment compared to placebo, plus selected focal comparisons of interest between licensed dose regimens, in each external target population using ML-NMR.

	External target population		
Contrast	PsoBest	PROSPECT	Chiricozzi 2019
ETN vs. PBO	1.68<br>(1.52, 1.85)	1.70<br>(1.48, 1.94)	1.76<br>(1.55, 1.98)
IXE Q2W vs. PBO	2.95<br>(2.79, 3.11)	3.00<br>(2.80, 3.23)	3.01<br>(2.81, 3.24)
IXE Q4W vs. PBO	2.78<br>(2.63, 2.94)	2.83<br>(2.63, 3.06)	2.84<br>(2.64, 3.07)
SEC 150 vs. PBO	2.32<br>(2.13, 2.51)	2.37<br>(2.13, 2.62)	2.38<br>(2.16, 2.62)
SEC 300 vs. PBO	2.73<br>(2.55, 2.91)	2.78<br>(2.55, 3.03)	2.79<br>(2.57, 3.02)
UST vs. PBO	2.26<br>(1.99, 2.53)	2.23<br>(1.94, 2.55)	2.26<br>(1.97, 2.56)
SEC 300 vs. IXE Q2W	−0.22<br>(−0.39, −0.07)	−0.22<br>(−0.39, −0.07)	−0.22<br>(−0.39, −0.07)
UST vs. IXE Q2W	−0.69<br>(−0.94, −0.43)	−0.77<br>(−1.01, −0.54)	−0.75<br>(−0.99, −0.52)
UST vs. SEC 300	−0.47<br>(−0.69, −0.25)	−0.55<br>(−0.78, −0.31)	−0.53<br>(−0.75, −0.31)

Table A.9: Estimated proportion of individuals achieving each PASI outcome on each treatment in each external target population with information on response rates, along with 95% Credible Intervals, using the ML-NMR model combining information from all PASI endpoints. For interpretability, these are given as inclusive probabilities (e.g. the probability of achieving 75% reduction or greater in PASI score).

	Treatment						
Population	Placebo	Etanercept	Ixekizumab Q2W	Ixekizumab Q4W	Secukinumab 150 mg	Secukinumab 300 mg	Ustekinumab
Chiricozzi 2019							
PASI 75	2.51 (1.10, 4.79)	38.73 (28.68, 49.38)	80.08 (73.84, 85.33)	75.17 (68.25, 81.55)	59.23 (52.08, 66.16)	73.62 (68.82, 78.27)	60.68 (48.06, 72.83)
PASI 90	0.45 (0.16, 1.00)	17.25 (10.86, 24.93)	57.25 (48.90, 65.28)	50.73 (42.26, 59.18)	33.41 (26.80, 40.43)	48.73 (42.79, 54.90)	34.58 (23.35, 47.35)
PASI 100	0.03 (0.01, 0.08)	4.00 (2.03, 6.82)	26.55 (19.90, 33.65)	21.46 (15.59, 28.25)	10.79 (7.52, 14.74)	19.96 (15.81, 24.55)	11.24 (5.93, 18.56)
PROSPECT							
PASI 75	3.45 (1.70, 6.10)	41.49 (32.86, 50.75)	82.56 (78.11, 86.47)	78.03 (72.74, 82.74)	62.93 (58.08, 67.77)	76.60 (74.47, 78.70)	65.13 (54.15, 75.41)
PASI 90	0.67 (0.27, 1.38)	19.33 (13.39, 26.21)	61.00 (54.40, 67.03)	54.62 (47.63, 61.35)	37.14 (32.15, 42.61)	52.65 (49.40, 55.79)	39.12 (28.33, 50.49)
PASI 100	0.05 (0.02, 0.13)	4.80 (2.76, 7.50)	29.98 (24.25, 35.99)	24.57 (19.17, 30.44)	12.87 (9.94, 16.30)	22.96 (20.18, 25.94)	13.76 (8.18, 20.93)

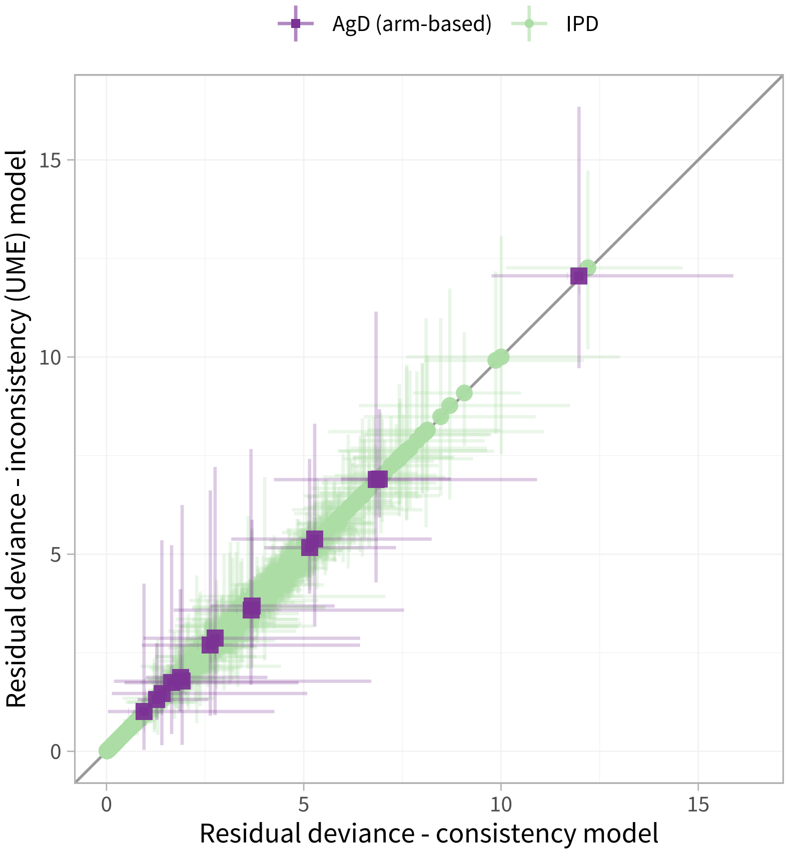

Figure A.1: Residual deviance contributions (posterior mean and 95% Credible Interval) under a fixed effect ML-NMR model assuming consistency, and under a fixed effect ML-NMR inconsistency (UME) model. Here, all of the posterior mean residual deviance contributions lie on the line of equality; all points fit equally well under either model.

A.4 Comparison with aggregate data NMA

For models to be comparable using DIC, we need the input data to be the same. The DIC values in Table 2 for the NMAs are therefore calculated considering the residual deviance of each IPD point separately (i.e. with the uncollapsed data, not data collapsed into summary counts as is usual), so that direct comparison can be made with the ML-NMR models. The collapsed and uncollapsed NMA models are mathematically equivalent, and the conclusions from model comparison between the NMA models are also the same since the residual deviance and DIC are equivalent up to a fixed constant.

The estimated heterogeneity standard deviation from the RE NMA without covariate adjustment was 0.09 (0.01, 0.24), which is small compared to the magnitude of the relative effects (Table A.4). The DIC values for the FE and RE NMA models without covariate adjustment were 8948.2 and 8947.5 respectively (see Table 2); there is little difference between these models, and we would choose the more parsimonious fixed effect model based on DIC alone. The DIC for an UME model which does not make the consistency assumption is 8950.1 which is a little higher than the FE consistency model, and a plot of the residual deviance contributions under each model (Figure A.2) shows that all data points are fit equally well under either model, altogether suggesting no evidence of inconsistency. However, despite a lack of evidence for between-study heterogeneity or inconsistency, the ML-NMR model has a much lower DIC of 8814.9. The ML-NMR model allows us to explain both between and within study variation, resulting in better fit and reduced uncertainty in contrast estimates across the study populations (Table A.4).

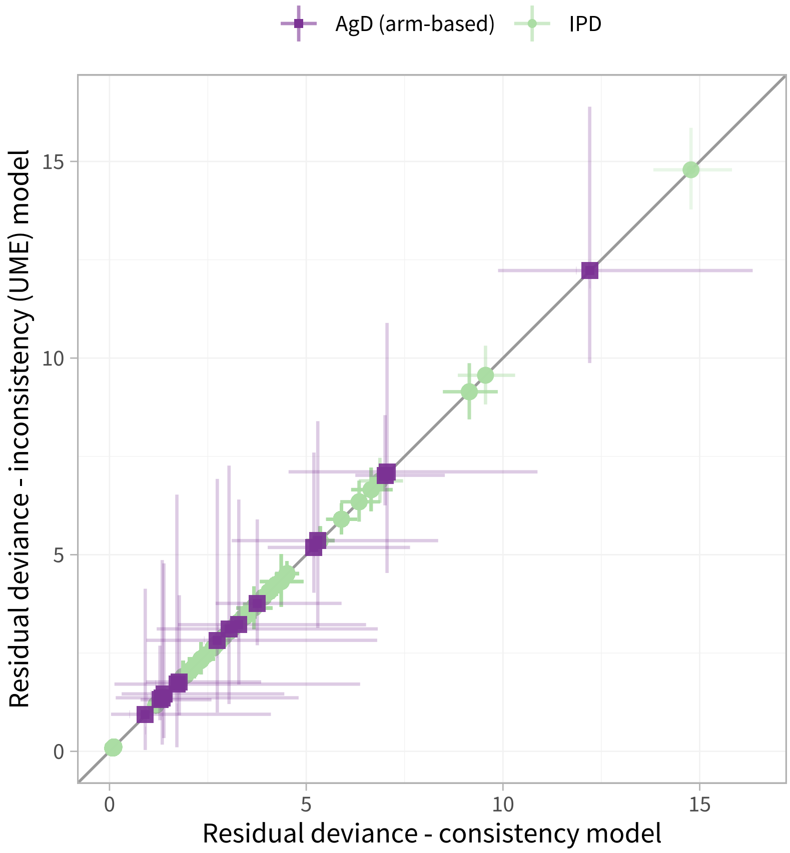

Figure A.2: Residual deviance contributions (posterior mean and 95% Credible Interval) under a fixed effect NMA model assuming consistency, and under a fixed effect NMA inconsistency (UME) model. Here, all of the posterior mean residual deviance contributions lie on the line of equality; all points fit equally well under either model.

A.5 Assessing the shared effect modifier assumption

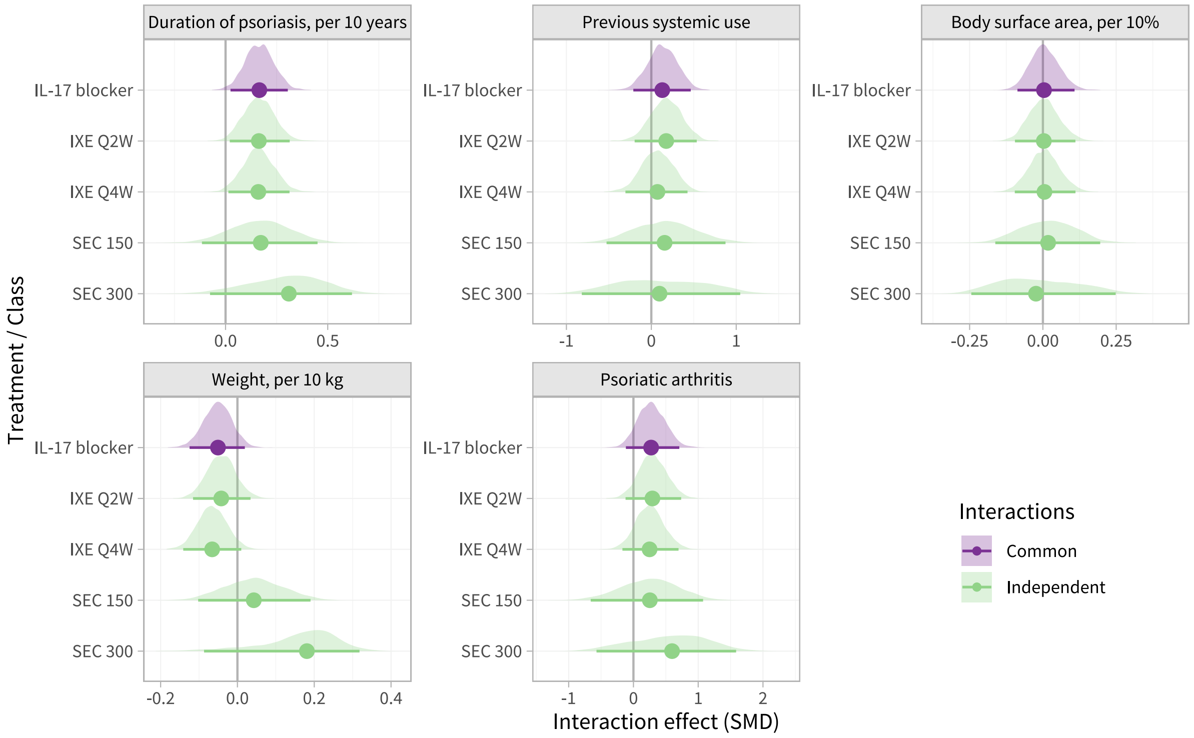

Figure A.3: Posterior estimates (median, 95% CrI, and density) of effect modifier interaction parameters from the ML-NMR model making the shared effect modifier assumption within the IL-17 blocker class for all covariates, and the ML-NMR models relaxing this to independent interactions for each covariate in turn.

Table A.10: Model fit statistics for the ML-NMR model making the shared effect modifier assumption within the IL-17 blocker class for all covariates, and the ML-NMR models relaxing this to independent interactions for each covariate in turn. p_D is a measure of the effective number of parameters. Residual deviance on 12,384 data points.

		Independent interactions for covariate				
	Common interactions	Duration of psoriasis	Previous systemics	Body surface area	Weight	Psoriatic arthritis
Residual deviance	8778.3	8780.9	8779.9	8780.9	8775.3	8780.6
p_D	36.6	38.9	38.6	38.7	39.6	38.5
DIC	8814.9	8819.9	8818.5	8819.6	8815.0	8819.1

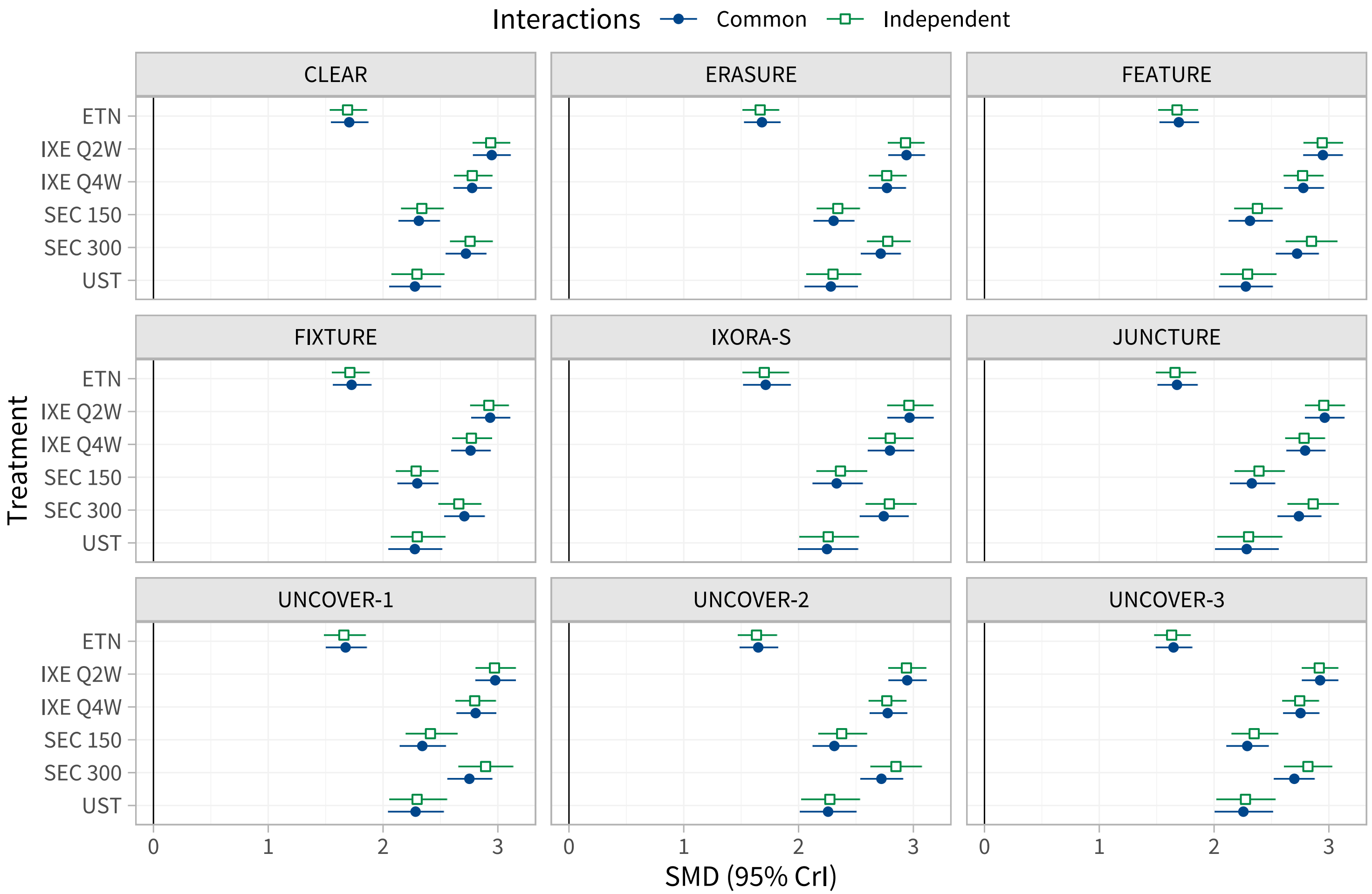

Figure A.4: Estimated population-average treatment effects (standardised mean differences) for each treatment vs. placebo in each study population, from the ML-NMR model making the shared effect modifier assumption within the IL-17 blocker class for all covariates, and the ML-NMR model relaxing this to independent interactions for weight.

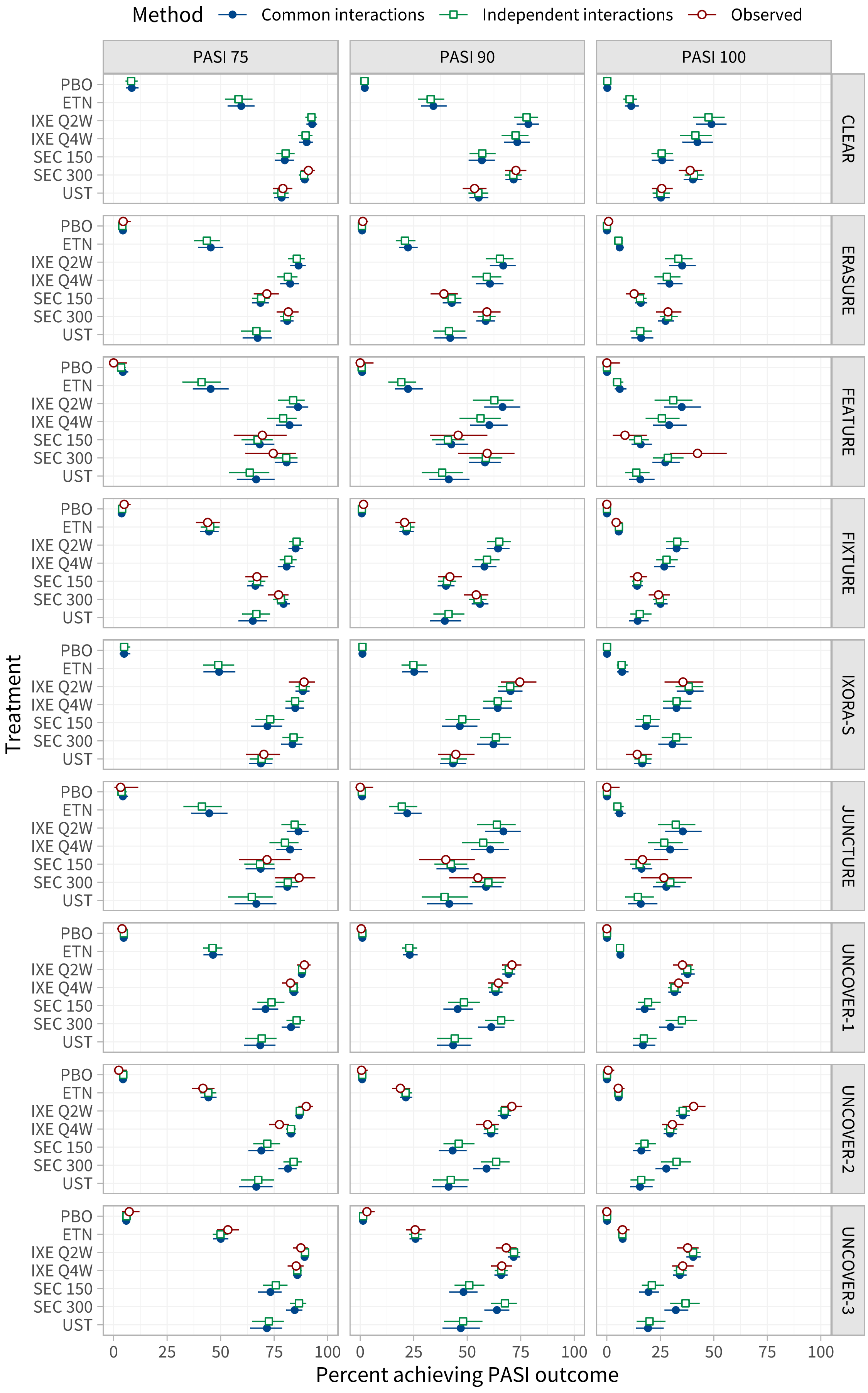

Figure A.5: Estimated percentage of individuals achieving each PASI endpoint on each treatment, in each study population, from the ML-NMR model making the shared effect modifier assumption within the IL-17 blocker class for all covariates, and the ML-NMR model relaxing this to independent interactions for weight.

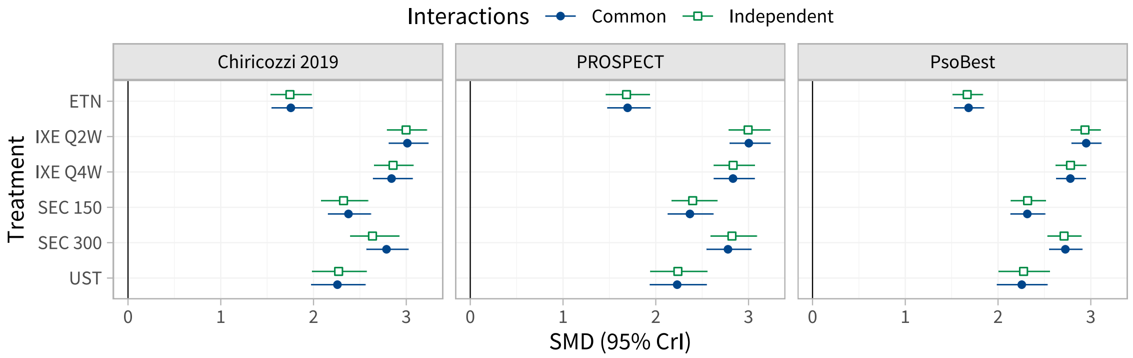

Figure A.6: Estimated population-average treatment effects (standardised mean differences) for each treatment vs. placebo in each external target population, from the ML-NMR model making the shared effect modifier assumption within the IL-17 blocker class for all covariates, and the ML-NMR model relaxing this to independent interactions for weight.

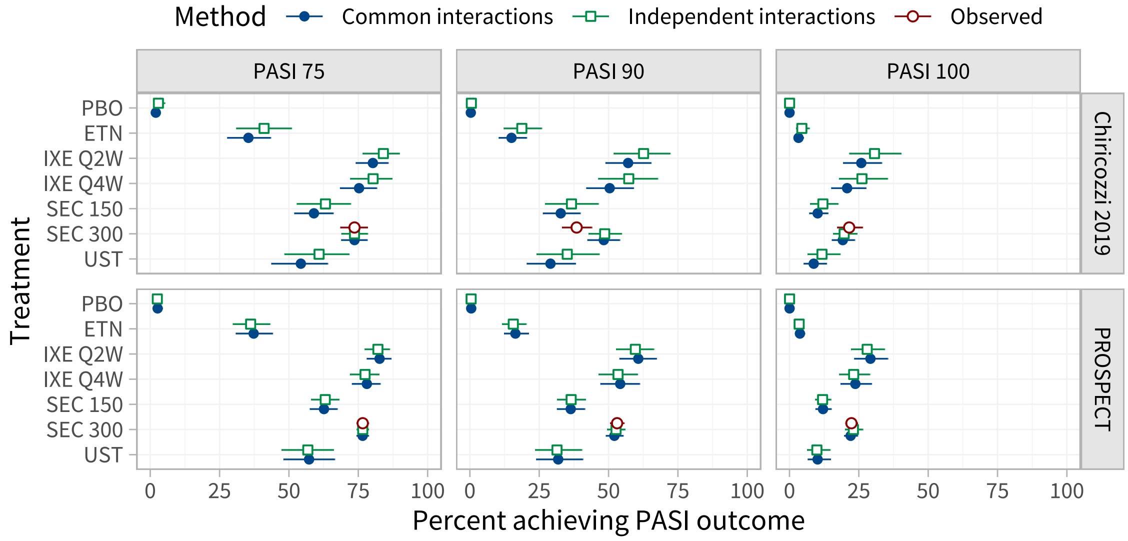

Figure A.7: Estimated percentage of individuals achieving each PASI endpoint on each treatment, in each external target population with information on response rates, from the ML-NMR model making the shared effect modifier assumption within the IL-17 blocker class for all covariates, and the ML-NMR model relaxing this to independent interactions for weight.
